# Noise constrains the evolution of call frequency contours in flowing water frogs: a comparative analysis in two clades

**DOI:** 10.1186/s12983-021-00423-y

**Published:** 2021-08-04

**Authors:** Longhui Zhao, Juan C. Santos, Jichao Wang, Jianghong Ran, Yezhong Tang, Jianguo Cui

**Affiliations:** 1 CAS Key Laboratory of Mountain Ecological Restoration and Bioresource Utilization and Ecological Restoration Biodiversity Conservation Key Laboratory of Sichuan Province, Chengdu Institute of Biology, Chinese Academy of Sciences, Chengdu, 610041 Sichuan China; 2 Ministry of Education Key Laboratory for Tropical Plant and Animal Ecology, College of Life Sciences, Hainan Normal University, Haikou, 571158 Hainan China; 3 Key Laboratory of Bio-Resource and Eco-Environment of Ministry of Education, College of Life Sciences, Sichuan University, Chengdu, 610065 Sichuan China; 4 Department of Biological Sciences, St. John’s University, Queens, NY 11439 USA

**Keywords:** Acoustic signals, Anurans, Ecological selection, Frequency-contour complexity, Noise

## Abstract

**Background:**

The acoustic adaptation hypothesis (AAH) states that signals should evolve towards an optimal transmission of the intended information from senders to intended receivers given the environmental constraints of the medium that they traverse. To date, most AAH studies have focused on the effect of stratified vegetation on signal propagation. These studies, based on the AAH, predict that acoustic signals should experience less attenuation and degradation where habitats are less acoustically complex. Here, we explored this effect by including an environmental noise dimension to test some AAH predictions in two clades of widespread amphibians (Bufonidae and Ranidae) that actively use acoustic signals for communication. By using data from 106 species in these clades, we focused on the characterization of the differences in dominant frequency (DF) and frequency contour (i.e., frequency modulation [FM] and harmonic performances) of mating calls and compared them between species that inhabit flowing-water or still-water environments.

**Results:**

After including temperature, body size, habitat type and phylogenetic relationships, we found that DF differences among species were explained mostly by body size and habitat structure. We also showed that species living in lentic habitats tend to have advertisement calls characterized by well-defined FM and harmonics. Likewise, our results suggest that flowing-water habitats can constrain the evolutionary trajectories of the frequency-contour traits of advertisement calls in these anurans.

**Conclusions:**

Our results may support AAH predictions in frogs that vocalize in noisy habitats because flowing-water environments often produce persistent ambient noise. For instance, these anurans tend to generate vocalizations with less well-defined FM and harmonic traits. These findings may help us understand how noise in the environment can influence natural selection as it shapes acoustic signals in affected species.

**Supplementary Information:**

The online version contains supplementary material available at 10.1186/s12983-021-00423-y.

## Background

Acoustic signals are important behavioural traits in species delimitation, reproductive isolation, and animal communication [1, 2]. Revealing the mechanisms that result in signal divergences is a relevant topic in the evolutionary ecology of animals that use acoustic signals. Several evolutionary mechanisms are major drivers of acoustic divergence, including neutral and mutation-order processes as well as natural and sexual selections [3]. Over the past few decades, the effects of the ecological (environmental) context and mate choice pressures on acoustic signals have been focal points of research in the bioacoustics of animals. Some evidence (particularly in anurans) suggests that neutral processes are also important for signal divergence [4]. To date, many empirical studies support the impact of sexual (e.g., mate choice) and natural (e.g., habitat structure) selection as drivers of the diversification of signals in anurans [5, 6]. However, few comparative analyses have focused on the evolution of acoustic signals as a consequence of adaptations to their environmental background among clades of anurans with statistical methods addressing the underlying phylogenetic context.

The environmental context and physical properties of the habitat can constrain acoustic transmission when long-distance sound communication is used [7]. According to the acoustic adaptation hypothesis (AAH), natural selection shapes acoustic signals in a way that maximizes their transmission distance and content integrity within the environment in which they are produced [8]. These predictions have been supported by evidence of sound signalling in birds and other vertebrates, where their vocalizations evolved to increase propagation and accurate information transmission in specific environments [8–10]. In this context, several principles are proposed according to the AAH that characterizes acoustic signals. For example, high-frequency and more frequency-modulated calls are favoured in open areas where little degradation of acoustic signals exists [11]. Signals, when produced closer to the ground, tend to have a frequency range over 500–1000 Hz, which provides them with better propagation properties near the ground [8, 12].

In the case of environmental noise, the AAH predicts that long-range communication in vertebrates should account for such signal degradation [13, 14]. For instance, animals seem to be able to change their vocalization characteristics to adjust or compensate for signal degradation in noisy environments. For example, mating calls in frogs and birds seem to evolve towards higher frequencies in noisy habitats to avoid masking [15, 16]. In the long term, some species might also be able to adapt or even evolve in response to noise exposure [11]. Several large-scale studies showed a strong correlation between dominant frequency (DF) and background noise, while temporal call features were not found to be shaped by the habitat conditions where the signaller was vocalizing [10, 17, 18]. Frequency modulation (FM) and harmonic traits might also be affected by noisy environments [10, 12]. However, whether long-term noisy environments can result in the evolution of FM properties and frequency contours towards optimal signal transmission is less known.

Anurans rely heavily on acoustics to send mating and territorial signals that convey information about the location of the signaller, aggression, and health condition (i.e., quality) to mates and competitors [19]. The habitat and life history of most frogs, e.g., short migratory ranges and strong reliance on sound to facilitate location by partners, have shaped the evolution of mating calls [20]. Interestingly, some frogs inhabit noisy environments that can obfuscate their acoustic communication. The mating calls of these taxa should evolve to compensate for any distortions, such as the distortions resulting from acoustic conditions in many torrential streams (i.e., overwhelmed by the noise produced by running water; [21]). In these habitats, most species evolved audio-visual or noise-adjusted acoustic signals, e.g., visual signals such as foot flagging and foot flashing or high-frequency calls [22–24]. Therefore, anurans with noise-adjusted signals are an excellent group for exploring the impacts of long-term noise exposure and adaptations of acoustic signals in species that inhabit noisy environments.

Stream habitats often have ecological contexts characterized by flowing water [25]. The sound derived from water rushing over rocks and other substrates can cause continuous background noise. The background noise in environments dominated by flowing water centres more energy in the low-frequency range [15, 26] and can pass long distances with less attenuation [27]. Therefore, habitats with flowing water should be noisier than still-water habitats (i.e., noisy environments vs. less noisy environments). For instance, a recent study showed that clades of ranid frogs living in flowing water habitats are exposed to higher background noise levels than other Ranidae clades [17]. Moreover, Goutte et al. [10] measured the ambient noise of the microhabitat of many anurans, and their data show that lotic and lentic habitats are acoustically different with regard to noise intensity (lotic habitats: 68.1 ± 7.6 dB; lentic habitats: 53.2 ± 7.3 dB).

Here, we investigate whether the flowing-water environment affects the evolution of the DF range, the presence or absence of harmonics, and FMs in two families of widespread anurans common in noisy environments, Bufonidae (toads) and Ranidae (pond frogs). Anurans use several kinds of acoustic signals, including advertisement, courtship, aggressive, and distress calls [28]. We focused only on advertisement calls, which are widely emitted by males and usually recorded by researchers. These signals can inform potential mates or competitors about the location of the signaller, identity, quality and territory ownership [28]. Environmental and morphological traits may have a complex influence on the evolution of acoustic signals [29]. However, these factors should be considered in conjunction with evolutionary history (phylogenies) if analyses of adaptation aim to elucidate relationships between habitats and call characteristics [10]. In this study, we used comparative methods and integrated potential confounding factors (i.e., temperature, body size and phylogenetic relationships) to explore the differences in acoustic signals between frogs living in flowing versus lentic habitats. In noisy environments, the structure of the frequency contour might not be easy to detect by receivers, yet the concentration of energy in a narrow frequency range can increase the detectability of the acoustic messages from signallers [30, 31]. Thus, we hypothesized that species that vocalize in noisy flowing-water habitats would have simpler calls (i.e., fewer harmonics and FMs) than species that have evolved in environments with quieter backgrounds.

## Methods

### Taxa studied

We focused on two globally distributed families of frogs: Bufonidae and Ranidae (628 species and 410 species, respectively). Both groups breed in ponds, lakes, and other lentic environments, as well as in lotic environments such as waterfalls, rivulets, and torrential streams [32]. Many taxa of these two families have significant variability in their body size, but most taxa have similar morphology and natural history [32]. Moreover, a torrent-dwelling lifestyle has evolved multiple times in the two families. For instance, ranids have at least six genera reproducing in streams [33], and their torrent-dwelling lifestyle was acquired via multiple independent evolutionary events [17]. Nevertheless, both families usually have advertisement calls with simple temporal structures (i.e., simple notes/pulses) and spectral properties (e.g., few harmonics per note). These acoustic properties make both groups ideal to explore the consequences of flowing-water (lotic) versus still-water (lentic) environmental constraints on advertisement call diversification and evolution.

### Data collection

We obtained call, morphology, and life history data from published accounts, AmphibiaWeb (https://amphibiaweb.org/), and expert personal communications on our target clades: Bufonidae and Ranidae. These data included spectral and temporal properties of advertisement calls, body size, habitat characterization, and noise levels where the calls were produced. Next, we describe the characteristics of these data.

Some frequency parameters of the advertisement call may be affected by temperature [34, 35]. For this reason, we included the environmental temperature in analyses associated with sound recordings to evaluate the effects of temperature on call frequency. We chose air or water temperature depending on the environmental medium that the animal inhabited during the sound recordings. We extracted the mean DF from the descriptions (99 species) and sound recordings (7 species). The mean value was determined if there was more than one datum available. We determined whether the advertisement calls had harmonics and whether the calls exhibited significant FM, such as those observed in *Rhinella magnussoni* [36] and *Babina daunchina* [37]. If calls showed harmonics, they were defined as harmonic vocalizations (1-present), or if they lacked harmonics, they were defined as nonharmonic vocalizations (0-absent). If the frequency change was prominent in a dominant frequency band (range over > 20% of the bandwidth), the FM variable was defined as 1-present. If not, the FM variable was considered as 0-absent.

These repertoires were obtained from spectrograms and determined to minimize errors associated with sound analyses where sharpness, time, and space resolution can hinder accurate identification. For example, we used this approach if the difference in window size altered the shape of frequency bands when performing fast Fourier transformation (FFT). Extensively fuzzy or degraded (i.e., low-quality) images of printed spectrograms without sound recordings were excluded from the analyses to assure the reliability of harmonic and frequency-modulated sweep characterizations. For those species with digitized sound files, calls were analysed from raw files derived from available online archives, kindly provided by other researchers or our own previous studies. In these cases, all spectrograms were automatically generated using the FFT method (window type: Blackmann-Harris) in Adobe Audition 3.0 software (Adobe Systems Inc., San Jose, CA, USA). In some cases, a correction was made if there was an inconsistency between the result from the reference and our analyses. All series of details about the sources can be seen in Additional file 1.

For body size, we used the male snout-vent length (SVL). In some cases, SVL reports are ranges, and we uniformly chose the maximum SVL, as this measurement correlates highly (i.e., 98% in anurans) with the mean SVL values [18]. For habitat characteristics, we recorded breeding sites for each taxon based on their exclusive use of standing (lentic) water or running (lotic) water (i.e., excluded taxa that utilize both). This approach allowed us to binarize species into two exclusive categories: (1) still-water habitat, or (2) flowing-water habitat. The location of calling (vocalizing) activity was separated from breeding sites (e.g., spawning grounds) in some species, and the habitat type was classified based on details of the calling location to avoid potential bias. Finally, we collected the noise levels of some species that called at the two habitat types (Additional file 2).

### Molecular phylogeny

We obtained acoustic information on 48 species from 16 genera of the family Bufonidae and 58 species from 17 genera of the family Ranidae. To reconstruct their phylogenetic relationships, we downloaded 298 sequences of 82 species from GenBank (Additional file 3). As outgroups, we used four sequences of a Leptodactylidae species and three sequences of a Rhacophoridae species. The molecular markers were 12S and 16S rRNA, chemokine receptor 4 (CXCR4), recombination activating protein 1 (RAG-1), and rhodopsin. These sequences were aligned using MEGA version 7.0 [38]. After the alignment, we partitioned the data and selected substitution models according to the Bayesian information criterion (BIC) by using PartitionFinder v2.1.1 [39]. Nuclear and mitochondrial genes were divided based on the proposed best partitioning strategy. The phylogenetic relationships were reconstructed via Bayesian inference with MrBayes version 3.2.22 [40]. The results of the Bayesian inference (Additional file 4) did not significantly differ from the reported phylogenies of Bufonidae and Ranidae [33].

### Data analyses

All statistical analyses were completed in R version 3.5.3. The Wilcoxon rank sum test was used to compare the differences in SVL and dominant frequency in still-water species and flowing-water species. Blomberg's *K* was used to evaluate the phylogenetic dependence of continuous data (i.e., DF and SVL) [41]. *K* varies from 0 to infinity and indicates the resemblance of closely related species under a Brownian motion model of signal evolution. Pagel’s λ was used to examine the phylogenetic dependence of discrete traits (i.e., habitat type, harmonic and FM) [42]. The DF and SVL showed a strong phylogenetic signal (see “Results”). We thus employed a phylogenetic generalized least squares (PGLS) method to analyse the influence of calling sites (i.e., still water/flowing water) on call DF. The PGLS model was run with the R package *caper* [43]. Call frequency may be correlated with body size and environmental temperature; thus, we included SVL, recorded temperature, and calling environment (i.e., flowing water or still water) as explanatory variables in the model. An interaction term ‘Environment x SVL’ was included to determine if calling environment and SVL influence call parameters. Prior to the analysis, DF and SVL data were *ln* transformed as in previous studies [17, 18]. Moreover, we set the λ values by maximum likelihood to optimize branch-length transformations in the model.

We tested whether calls with or without significant FM or harmonics had phylogenetic signals. For this purpose, we used the ‘fitDiscrete’ function of the R package *geiger* [44]. We compared two models by forcing Pagel’s λ to be equal to 0 (no signal) versus a model with λ as a free parameter. Then, we calculated the likelihood ratio between the two models and determined its significance by chi-squared approximation. Our results indicated no significant phylogenetic signal. Therefore, we used Fisher's exact test (only the FM data in Bufonidae) or Pearson's chi-squared test (all others) to compare the number of species that possess such traits (i.e., FM and harmonics) between still-water and flowing-water environments. Finally, we calculated the mean SPLs of still-water species and flowing-water species.

## Results

### Effect of habitat, body size (SVL), and temperature on dominant frequency (DF)

We found that flowing-water species (*N* = 45) had a mean SVL of 45.2 ± 24.5 cm (range 18–138 cm) and produced advertisement calls with a mean DF of 4.59 ± 3.99 kHz (0.72–19 kHz), while still-water species (*N* = 61) had an average SVL of 66.8 ± 21.7 cm (25.5–126 cm) and generated advertisement calls with an average DF of 1.69 ± 0.84 kHz (0.35–4.23 kHz). Further analyses showed that the DF of the calls and the SVL were significantly different between still-water and flowing-water species (DF: *W* = 468, *P* < 0.001; SVL: *W* = 1945.5, *P* < 0.001). In anurans, DF and SVL have been found to show phylogenetic signals across clades and families [10, 18]. In the focal groups, i.e., Bufonidae and Ranidae, we found that both DF (*N* = 78, *K* = 0.32, Z variance =  − 3.03, *P* < 0.001) and SVL (*N* = 78, *K* = 0.22, Z variance =  − 2.81, *P* < 0.001) showed a strong phylogenetic signal.

**Fig. 1:**
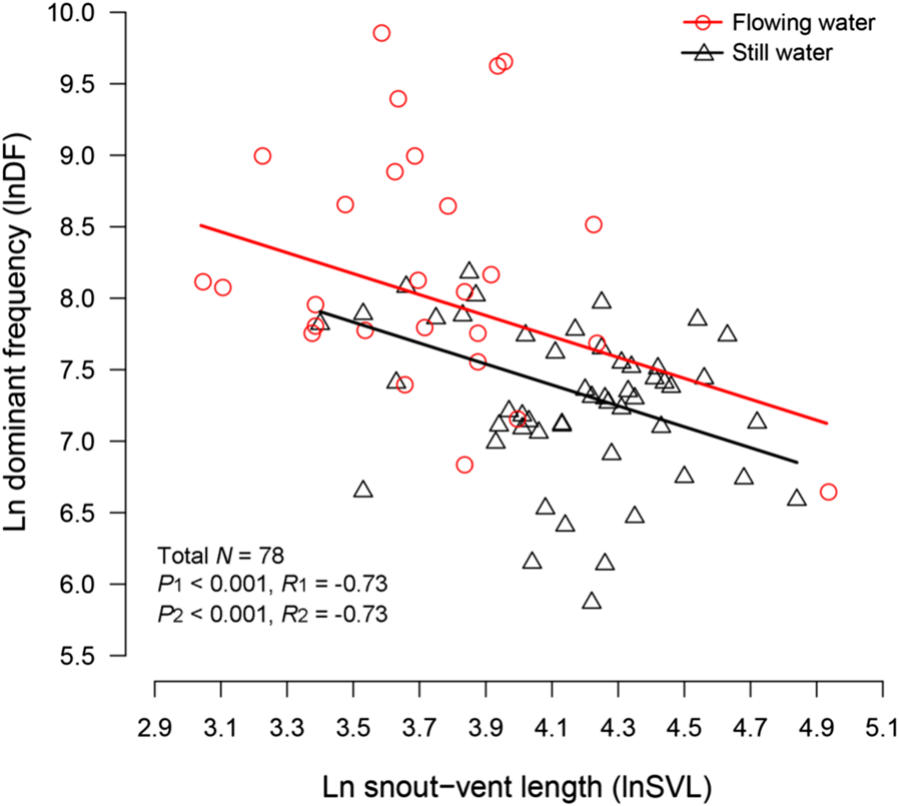
Relationship between dominant frequency (DF) and snout-vent length (SVL) for flowing-water species and still-water species. Regressions from the PGLS model are represented by solid lines

The results of the PGLS analyses suggested that the environmental temperature during sound recordings had no significant effect on the DF of advertisement calls (*N* = 56, *F* = 0.013, *P* = 0.911). In contrast, we observed that calling environments, i.e., flowing or still water, (*N* = 78, *F* = 9.42, *P* < 0.01) and body size (*N* = 78, *F* = 20.25, *P* < 0.001) had a significant influence on the DF of advertisement calls (Table 1; Fig. 1). Furthermore, there was no obvious interaction between the calling environment and body size (Table 1; Fig. 1). As seen in Fig. 1, the observed negative association between body size and DF and the slope of this association were the same for species calling from both environments. For the same body sizes, the DF was higher for species in habitats with flowing water than for species in habitats with still water.

**Table 1:** Effects of calling environment and body size (SVL) on call dominant frequency (DF) (ANOVA results for the PGLS model)

Source	*df*	Sum Sq	Mean Sq	*F*	*P*
Environment	1	29.429	29.429	9.4216	0.003
SVL	1	63.266	63.266	20.2542	< 0.001
Environment × SVL	1	7.368	7.368	2.3587	0.129
Residuals	74	231.145	3.124	–	–

### Constraints of flowing-water (noisy) environments on the frequency contours of calls

**Fig. 2:**
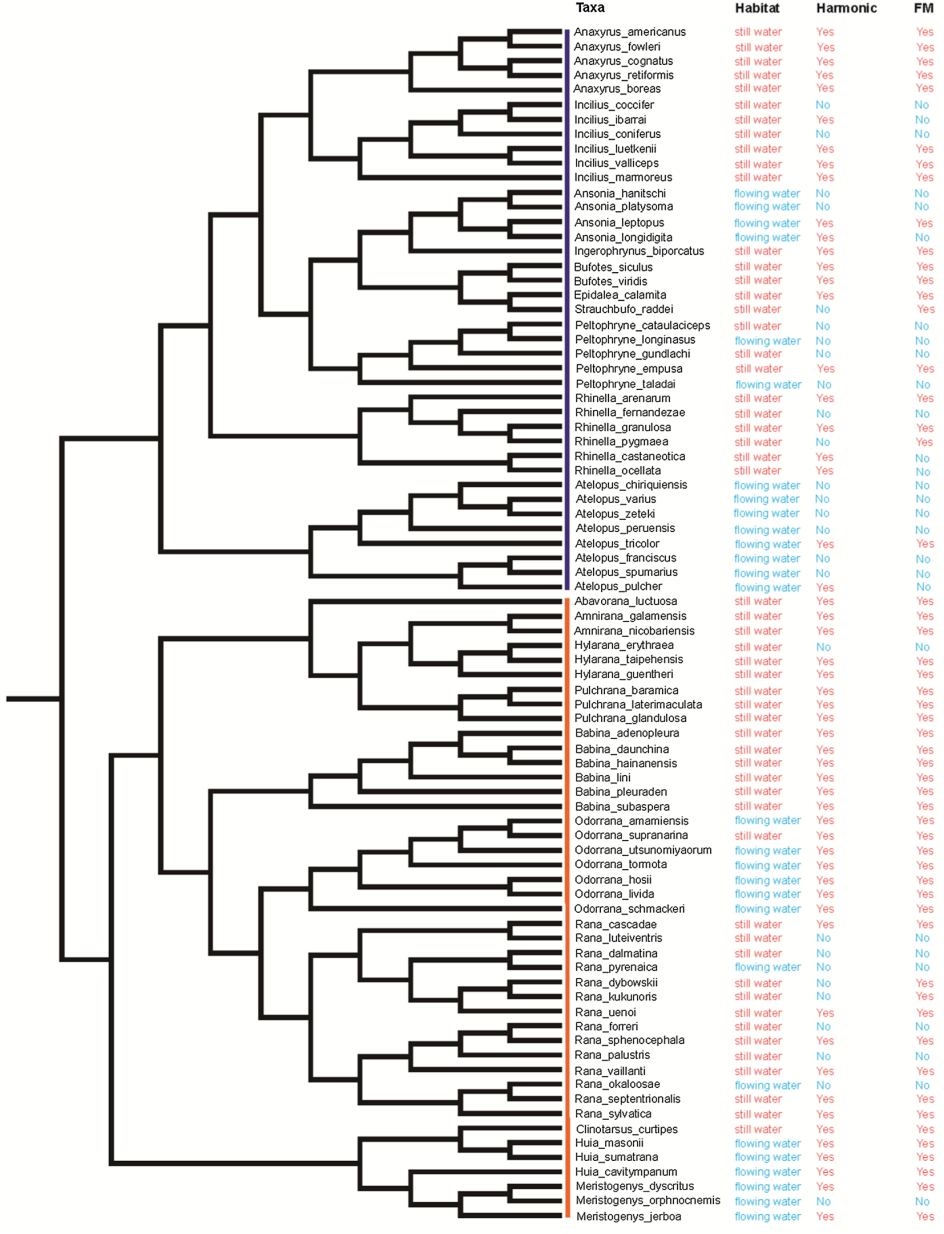
Phylogeny of Bufonidae and Ranidae species included in Pagel’s λ model, with corresponding habitat and call characters. Bars next to the taxa names indicate Bufonidae (blue) and Ranidae (orange). Call characters reflect whether each taxon has harmonic and conspicuous FM

**Fig. 3:**
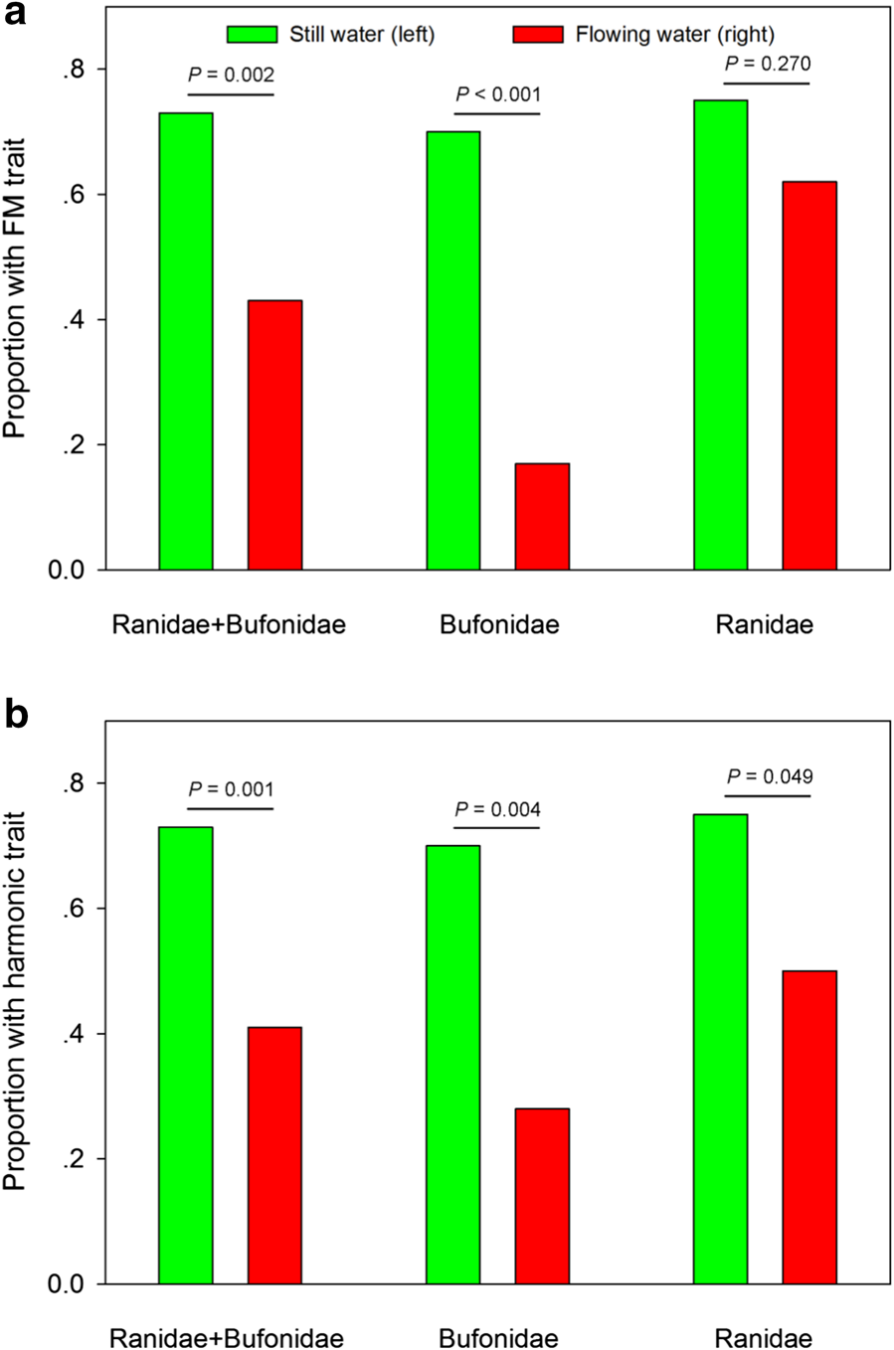
Comparisons of frequency modulation (FM) and harmonic traits between still-water species and running-water species across Ranidae and/or Bufonidae. **a** Proportion of species with FM patterns in Ranidae and/or Bufonidae. **b** Proportion of species with harmonic patterns in Ranidae and/or Bufonidae

Call and habitat characteristics were mapped on the phylogenetic tree (Fig. 2); these traits included habitat types (i.e., flowing water vs. still water) and frequency-contour traits (i.e., harmonics and FM). Pagel’s λ results suggested that habitat types of Ranidae and Bufonidae showed a strong phylogenetic signal (*N* = 82, λ ~ 1.0 with *P* < 0.001; Table 2). In contrast, the presence or absence of harmonics and the presence or absence of significant FM showed no phylogenetic signal (*N* = 82, *P* > 0.05; Table 2). For those nonphylogenetic signals, we did not adjust for phylogeny, as suggested by analyses using simulated data [45]. Therefore, we compared the relationships between habitat and frequency-contour traits directly, and the results of all comparisons are shown in Fig. 3.

**Table 2:** Phylogenetic dependence of habitat type (still/flowing water), harmonic and frequency modulation (FM) traits (results for the Pagel’s λ model)

Trait	*λ*	logL	logL0	Likelihood ratio	*P*
Environment	0.99	− 35.57	− 53.13	3.511951e+01	< 0.001
Harmonic	0.00	− 48.77	− 48.77	7.718751e−08	1.000
FM	0.98	− 46.77	− 48.23	2.913272e+00	0.088

Our results indicate that vocalizations of Bufonidae and Ranidae taxa differ in their FM characteristics between still-water versus running-water habitats. For both groups, we found significant differences in the proportion of species with prominent FM (Fig. 3a; Table 3). Moreover, the proportion of species that produced harmonics also differed significantly in flowing-water versus still-water habitats (Fig. 3b; Table 3). Further support for the above results was provided by separate analyses of both families. In Bufonidae, we found that the proportion of species characterized by prominent FM (Fig. 3a; Table 3) or harmonic performance (Fig. 3b; Table 3) was significantly higher in still-water environments than in flowing-water environments. In Ranidae, we obtained a similar result for the proportion of species that produced harmonics (Fig. 3b; Table 3). The proportion of species with prominent FM, however, was not significantly different between the two environments (Fig. 3a; Table 3), although still-water environments contained more species with this trait than flowing-water environments (24/32 vs. 16/26).

**Table 3:** The number of total species, still-water species (SW), still-water species that have or not have prominent frequency modulation (SW FM vs. SW NonFM) and harmonic (SW H vs. SW NonH), flowing-water species (FW), flowing-water species that have or not have prominent frequency modulation (FW FM vs. FW NonFM) and harmonic (FW H vs. FW NonH) across Ranidae or Bufonidae

Family	Total	SW	SW	SW	SW	SW	FW	FW	FW	FW	FW
			NonFM	FM	NonH	H		NonFM	FM	NonH	H
Ranidae + Bufonidae	106	62	17	45	17	45	44	25	19	26	18
Bufonidae	48	30	9	21	9	21	18	15	3	13	5
Ranidae	58	32	8	24	8	24	26	10	16	13	13

## Discussion

In two representative families of anurans (i.e., Bufonidae and Ranidae), frogs that vocalize in flowing-water habitats tend to produce advertisement calls with higher DF than frogs in lentic habitats, and this difference can be explained by differences in body size and the acoustic environment (flowing water vs. still water). However, our regression models support that there are no significant interactions between the two predictors. Likewise, we show that flowing-water environments are an important constraint on prominent FM traits and harmonic performance of advertisement calls. Our results seem to be in agreement with AAH predictions derived from environmental noise because flowing-water habitats are often characterized by continuous background noise.

### DF evolution in response to environmental noise

The AAH predicts that ecological selection favours acoustic signals with higher frequency ranges if the environment has persistent low-frequency noise, such as running water habitats [8]. Such a prediction has not been thoroughly evaluated in a large phylogenetic context whose inertia may act in different directions with natural selection and can induce analytical biases when it is excluded [17]. Recently, contradictory results were found in two phylogenetic studies that compared call DF between streamside breeding and other frog species. Vargas-Salinas and Amézquita [21] found that the call frequency of five families (e.g., Bufonidae and Centrolenidae) was not significantly different based only on habitat type, while Röhr et al. [18] found that stream-dwelling species of 31 families (e.g., Ranidae and Hylidae) had higher call frequencies than non-stream dwelling species. These results imply that the evolution of amphibian advertisement calls may be influenced by habitat type as well as other factors, such as natural history. In this study, we focused on Ranidae, in which four torrent-dwelling groups breed in fast-flowing streams. Our other study group was Bufonidae, which is characterized by most species having similar morphology and natural history. By taking into account body size, environmental temperature, and phylogenetic relationships, we found that the DF was higher for species in habitats with flowing water than for species in habitats with still water (Fig. 1). Our results are consistent with a recent phylogenetic analysis focused on Ranidae that used decibel values of noise levels to assess the potential limitations of background sound on acoustic signals [10].

Natural selection favours small body size in anurans living in flowing-water habitats [21]. One bio-acoustical consequence of having a smaller vibrating apparatus (e.g., vocal cords in frogs) is that the advertisement call frequency would be higher, i.e., the call frequency is negatively correlated with body size in the animals [46]. Our results support this general trend across our frog species from both flowing-water and still-water environments (Fig. 1), which is evidenced by their body size and DF relationships with habitat. Therefore, both morphological traits and habitat type contribute to explaining the considerable frequency difference between torrent and lentic species in our study.

Habitat structure is considered an important factor shaping acoustic signal evolution, and researchers have found that many animals can design their call frequency to optimize signal transmission that matches their own habitats (e.g., [9, 13]). In this context, mating call evolution can also be constrained by morphological characteristics, particularly body size [47, 48]. However, the roles of habitat and body size vary in different vertebrate groups. For instance, in mammals, a report on Felidae [9] suggested that open habitats favour lower call frequency than dense habitats, while body size had no significant influence. Studies on the largest family of songbirds (i.e., Thraupidae) showed that body mass had the greatest impact on vocal displays and that signalling environments did not play an important role in acoustic evolution [48]. However, a meta-analysis across birds found that both habitat structure and body size only weakly predicted acoustic characteristics [49]. Such studies often focus on the effects of vegetation on signal propagation and do not explore the effect of noise. Here, we added this missing noise dimension to the AAH framework, and we revealed how strongly torrential environments impact acoustic signals and body size.

As described in the Introduction, some researchers have shown effectively that lotic habitats produce more noise than lentic habitats. Analysing the environmental noise data of 36 published frog species obtained a similar result in this study (Additional file 2). We show that the level of noise in the background when these frogs are called in calm water (lake, pond, etc.) ranges from 43.8 to 71.5 dB (mean: 54.0 ± 7.1 dB). In contrast, noise associated with running water (stream, waterfall, etc.) is in the range of 48.8–81.8 dB (mean: 67.8 ± 7.6 dB). Moreover, in our study, most taxa that inhabit flowing-water environments are described to communicate in very noisy, torrential and fast-flowing lotic habitats (e.g., [16, 23, 50]). Thus, our results for lotic and lentic environments could be included in the noise dimension, although it might be a limitation to interpret the specific environment as a proxy for noise level.

### Frequency contour evolution in response to environmental noise

Advertisement calls carry information about the signaller condition in the frequency domain of its advertisement call. To our knowledge, most research on ecological characterizations of anuran acoustic spectral properties is restricted primarily to a few features, such as DF, frequency range and bandwidth. Likewise, FM patterns of frequency contours might reveal identity [51], orientation [52] and other signaller conditions. However, certain changes in signals are more difficult to detect and recognize in complex environments. In our study, we analysed other characteristics of advertisement calls, such as harmonic contents and FM patterns. We show that lentic frogs have more harmonic performance and a more prominent FM pattern than more acoustically restricted flowing-water frogs, which might need to signal within noisy environments.

Natural selection could result in changes in FM of the signal to optimize transmission in specific habitats. For instance, reports on birds and frogs show that a rapid and higher proportion of FM should be favoured in open habitats than in closed habitats [8, 10, 12, 53, 54]. Similarly, the AAH literature predicts that acoustic signals with less pronounced FMs are favoured in noisy environments to facilitate their propagation and reduce their degradation compared with quiet environments [12, 53]. A recent report on anurans deviates from such a prediction and shows that DF modulation is correlated with noise, suggesting that larger DF changes would result in some of the signal components being detuned from the noise frequency range [10]. Our FM results, however, suggest that anurans may facilitate communication amid flowing-water noise by narrowing the range of frequency contours.

FM can be ascending, 'v' shaped, descending and even measured quantitatively based on its slope [55]. In the present study, our acoustic and environmental data are collected mainly from published literature and databases, which imposes a limitation on some frequency measurements, such as FM range (i.e., max frequency-min frequency) and type. More works about such complex information are important to reveal how the evolution of FM characteristics is shaped by different habitats.

In this study, we found that the evolution of harmonics was constrained in flowing-water environments. We consider that one possible explanation for the observed frequency-contour patterns is a result of selection in the detectability of vocal signals and enhancement by concentrating energy in a relatively narrow frequency range [30, 31]. In this case, the signals evolve based on selective pressures by receivers to localize or detect acoustic signals [27], which can be adaptive when background noise overlaps with conspecific signals [56]. The observed simplification of the signals could also be explained as a proximate cause, where simplifying signals decreases the amount of information carried in a noisy environment without loss of fidelity [57]. Such modification has already been found in marine mammals. For instance, Beluga whales (*Delphinapterus leucas*) and bottlenose dolphins (*Tursiops truncatus*) emit calls with less FM when boat noise is louder [57, 58].

Similar to flowing water, anthropogenic noise pollution can interfere with signal transmission and degrade its fidelity as a result of the introduction of more low-frequency components [57, 59]. More research is needed to assess whether or how increasing anthropogenic noise can influence the evolution of frequency contours as well as the potential fitness of these species. Given that more complex signals are often preferred by females [60–62], the constraint on harmonics and frequency contours generated under noisy pollution may reduce the information transmitted by acoustic signals [63] and influence female mate choice.

## Conclusions

Here, we show that flowing-water frogs have smaller body sizes than still-water frogs in Bufonidae and Ranidae, and the DF difference between the two habitats (i.e., higher DF in torrent species) is significantly explained by the environment as well as by body size. To our knowledge, for the first time, we show that long-term noise exposure may constrain the evolution of frequency-contour complexity in anurans towards less complex calls with fewer harmonics and lower FM. Such constraints might have an important influence on sexual selection as well as on the divergence of acoustic signals. Our finding also provides implications for the adaptive evolution of animals that are dealing with noise pollution derived from human activities.

## Supplementary Information

**Additional file 1**: Table S1. Name, habitat type, body size, call characteristic, recording temperature and reference for all sampled species.

**Additional file 2**: Table S2. Name, sound pressure levels (SPLs), habitat types and data sources for all species.

**Additional file 3**: Table S3. GenBank accession numbers for all sampled species and outgroup *Buergeria buergeri*.

**Additional file 4**: Figure S1. Phylogenetic relationships of all species based on two mitochondrial genes and three nuclear genes.

## Data Availability

The dataset supporting the conclusions of this article is included in the supplementary information files.
