# Supplementary material for: Noise constrains the evolution of call frequency contours in flowing water frogs: a comparative analysis in two clades
Source: Front Zool. 2021 Aug 4;18:37. doi: 10.1186/s12983-021-00423-y (PMC8336270; doi:10.1186/s12983-021-00423-y)
Supplement: Supplementary file 1 — Additional file 1: Table S1. Name, habitat type, body size, call characteristic, recording temperature and reference for all sampled species. [file 12983_2021_423_MOESM1_ESM.docx]

| **Taxa** | **Breeding site** | **SVL (mm)** | **DF (Hz)** | **Harmonic** | **FM sweeps** | **Note/Pulse type** | **Call type** | **Temperature (℃)** | **Reference** |
| --- | --- | --- | --- | --- | --- | --- | --- | --- | --- |
| **Bufonidae** |  |  |  |  |  |  |  |  |  |
| *Anaxyrus americanus* | still water | 85 | 1650 | YES | YES | 1 | 1 | 19.1 | ([Zweifel 1968](#_ENREF_110)) (https://amphibiaweb.org/search/index.html) |
| *Anaxyrus boreas* | still water | 108 | 845 | YES | YES | 1 | 1 | ----- | (https://amphibiaweb.org/search/index.html) |
| *Anaxyrus cognatus* | still water | 103 | 2307 | YES | YES | 1 | 1 | 18 | ([Sullivan 1983](#_ENREF_98); [Cocroft & Ryan 1995](#_ENREF_23)) (https://amphibiaweb.org/search/index.html) |
| *Anaxyrus fowleri* | still water | 74.5 | 1900 | YES | YES | 1 | 1 | 19.5 | ([Zweifel 1968](#_ENREF_110)) (https://amphibiaweb.org/search/index.html) |
| *Anaxyrus retiformis* | still water | 47 | 3583 | YES | YES | 1 | 1 | 24 | ([Bogert 1962](#_ENREF_9)) |
| *Ansonia hanitschi* | flowing water | 32 | 5700 | NO | NO | 1 | 1 | 21 | ([Malkmus *et al.* 2002](#_ENREF_66)) |
| *Ansonia leptopus* | flowing water | 40 | 3364 | YES | YES | 2 | 2 | 18.5 | ([Matsui 1982a](#_ENREF_68); [Wood Jr. *et al.* 2008](#_ENREF_104)) (http://www.frogsofborneo.org/index.php/bufonidae/129-bufonidae/ansonia/leptopus) |
| *Ansonia longidigita* | flowing water | 50 | 3500 | YES | NO | 1 | 1 | 22 | ([Malkmus *et al.* 2002](#_ENREF_66)) |
| *Ansonia platysoma* | flowing water | 25 | 8000 | NO | NO | 1 | 1 | 24 | ([Malkmus *et al.* 2002](#_ENREF_66)) |
| *Atelopus chiriquiensis* | flowing water | 34 | 2374 | NO | NO | 1 | 1 | 18 | ([Jaslow 1979](#_ENREF_43); [Savage 2002](#_ENREF_88)) |
| *Atelopus franciscus* | flowing water | 21 | 3320 | NO | NO | 1 | 1 | ----- | ([Lescure & Marty 2000](#_ENREF_60); [Boistel *et al.* 2011](#_ENREF_10)) |
| *Atelopus peruensis* | flowing water | 38.5 | 1625 | NO | NO | 1 | 1 | 15 | ([Lötters *et al.* 1999](#_ENREF_53); [Coloma *et al.* 2000](#_ENREF_24)) |
| *Atelopus pulcher* | flowing water | 29.3 | 2429 | YES | NO | 2 | 1 | 23 | ([Hödl 1990](#_ENREF_36); [Lötters *et al.* 2002](#_ENREF_54)) |
| *Atelopus spumarius* | flowing water | 29.4 | 2832 | NO | NO | 1 | 1 | 27 | ([Cocroft *et al.* 1990](#_ENREF_22)) |
| *Atelopus tricolor* | flowing water | 22.3 | 3210 | YES | YES | 1 | 1 | 26.2 | ([Lötters *et al.* 1999](#_ENREF_53)) (https://amphibiaweb.org/search/index.html) |
| *Atelopus varius* | flowing water | 41 | 2409 | NO | NO | 1 | 1 | ----- | ([Cocroft *et al.* 1990](#_ENREF_22)) |
| *Atelopus zeteki* | flowing water | 48 | 1898 | NO | NO | 1 | 1 | 24.8 | ([Cocroft *et al.* 1990](#_ENREF_22)) (https://amphibiaweb.org/search/index.html) |
| *Blythophryne beryet* | still water | 25.5 | 2900 | NO | YES | 1 | 1 | ----- | ([Chandramouli *et al.* 2016](#_ENREF_17)) |
| *Bufotes siculus* | still water | 86.6 | 1600 | YES | YES | 1 | 1 | 16 | ([Stöck *et al.* 2008](#_ENREF_93); [Lo Valvo & Giacalone 2013](#_ENREF_65)) |
| *Bufotes viridis* | still water | 71.5 | 1434 | YES | YES | 1 | 1 | 17.5 | ([Giacoma *et al.* 1997](#_ENREF_34); [Castellano *et al.* 1999](#_ENREF_16)) (https://amphibiaweb.org/search/index.html) |
| *Epidalea calamita* | still water | 71 | 1477 | YES | YES | 1 | 1 | 12 | ([Arak 1988](#_ENREF_3); [Diego-Rasilla & Luengo 2004](#_ENREF_29)) (https://amphibiaweb.org/search/index.html) |
| *Incilius coccifer* | still water | 64.4 | 2403 | NO | NO | 1 | 1 | 26.7 | ([McDiarmid & Foster 1981](#_ENREF_76); [Mendelson III *et al.* 2005](#_ENREF_77)) |
| *Incilius coniferus* | still water | 72 | 1000 | NO | NO | 1 | 1 | 24 | ([Porter 1966](#_ENREF_81)) (https://amphibiaweb.org/search/index.html) |
| *Incilius ibarrai* | still water | 82.4 | 1700 | YES | NO | 1 | 1 | 19.5 | ([Porter 1966](#_ENREF_81); [Mendelson III *et al.* 2005](#_ENREF_77)) |
| *Incilius luetkenii* | still water | 96 | 1700 | YES | YES | 1 | 1 | 25 | ([Porter 1966](#_ENREF_81); [Savage 2002](#_ENREF_88)) |
| *Incilius marmoreus* | still water | 83 | 1825 | YES | YES | 1 | 1 | 26 | ([Porter 1966](#_ENREF_81); [Suazo-Ortuño *et al.* 2007](#_ENREF_97)) |
| *Incilius valliceps* | still water | 77.8 | 1480 | YES | YES | 1 | 1 | 22.5 | ([Sullivan & Wagner Jr 1988](#_ENREF_99); [Wagner Jr & Sullivan 1995](#_ENREF_103)) (https://amphibiaweb.org/search/index.html) |
| *Ingerophrynus biporcatus* | still water | 70 | 2100 | YES | YES | 1 | 1 | ----- | ([Kurniati *et al.* 2010](#_ENREF_51)) (https://amphibiaweb.org/search/index.html) |
| *Ingerophrynus quadriporcatus* | still water | ----- | 1012 | YES | YES | 2 | 2 | ----- | (https://amphibiaweb.org/search/index.html) |
| *Nectophrynoides asperginis* | flowing water | 18 | 4071 | YES | YES | highly variable | highly variable ----- | | ([Arch *et al.* 2011](#_ENREF_5)) (https://amphibiaweb.org/search/index.html) |
| *Nectophrynoides tornieri* | still water | 28 | 3120 | YES | YES | 1 | 1 | 17.5 | ([Starnberger *et al.* 2011](#_ENREF_94)) (https://amphibiaweb.org/search/index.html) |
| *Parapelophryne scalpta* | flowing water | 23 | 3444 | NO | NO | 1 | 1 | 21 | ([Fei *et al.* 2012](#_ENREF_30); [Sun 2017](#_ENREF_101)) |
| *Peltophryne cataulaciceps* | still water | ----- | 4228 | NO | NO | 1 | 1 | 23 | ([Alonso & Rodríguez 2003](#_ENREF_1)) (https://amphibiaweb.org/search/index.html) |
| *Peltophryne empusa* | still water | 76 | 1549 | YES | YES | 1 | 1 | 25.4 | ([Schwartz 1972](#_ENREF_89); [Alonso & Rodríguez 2003](#_ENREF_1)) (https://amphibiaweb.org/search/index.html) |
| *Peltophryne gundlachi* | still water | 34 | 2669 | NO | NO | 1 | 1 | 25.4 | ([Schwartz 1972](#_ENREF_89); [Alonso & Rodríguez 2003](#_ENREF_1)) (https://amphibiaweb.org/search/index.html) |
| *Peltophryne longinasus* | flowing water | 29 | 2329 | NO | NO | 1 | 1 | 22 | ([Schwartz 1972](#_ENREF_89); [Alonso & Rodríguez 2003](#_ENREF_1)) (https://amphibiaweb.org/search/index.html) |
| *Peltophryne taladai* | flowing water | 138 | 768 | NO | NO | 1 | 1 | 24.2 | ([Alonso & Rodríguez 2003](#_ENREF_1)) (https://amphibiaweb.org/search/index.html) |
| *Phrynoidis juxtasper* | flowing water | 138 | 718 | NO | NO | 2 | 2 | 26.5 | ([Inger 1964](#_ENREF_40); [Matsui 1982a](#_ENREF_68); [Lee & Stuebing 1990](#_ENREF_57)) |
| *Rentapia hosii* | flowing water | ----- | 1119 | NO | NO | 1 | 1 | ----- | (https://amphibiaweb.org/search/index.html) |
| *Rhinella arenarum* | still water | 112 | 1248 | YES | YES | 1 | 1 | 22 | ([Straneck *et al.* 1993](#_ENREF_95); [Salas *et al.* 1998](#_ENREF_86)) (https://amphibiaweb.org/search/index.html) |
| *Rhinella castaneotica* | still water | 37.7 | 1650 | YES | NO | 1 | 1 | 26 | ([Köhler & Lötters 1999](#_ENREF_45)) (https://amphibiaweb.org/search/index.html) |
| *Rhinella fernandezae* | still water | ----- | 2400 | NO | NO | 1 | 1 | ----- | ([Salas *et al.* 1998](#_ENREF_86)) |
| *Rhinella granulosa* | still water | 70 | 2906 | YES | YES | 1 | 1 | 28 | ([Lima *et al.* 2006](#_ENREF_61); [Sao-Pedro *et al.* 2011](#_ENREF_87)) (https://amphibiaweb.org/search/index.html) |
| *Rhinella magnussoni* | still water | 45.3 | 2259 | YES | YES | 1 | 1 | 26 | ([Lima *et al.* 2007](#_ENREF_62)) |
| *Rhinella ocellata* | still water | 53 | 1352 | YES | NO | 1 | 1 | ----- | ([Caldwell & Shepard 2007](#_ENREF_15)) |
| *Rhinella pygmaea* | still water | 42.7 | 2593 | NO | YES | 3 | 1 | 24.9 | ([Narvaes & Rodrigues 2009](#_ENREF_79); [de Carvalho *et al.* 2013](#_ENREF_28)) (https://amphibiaweb.org/search/index.html) |
| *Sclerophrys pardalis* | still water | 103.5 | 650 | NO | NO | 1 | 1 | ----- | ([Cherry & Francillon-Vieillot 1992](#_ENREF_19); [Cherry & Grant 1994](#_ENREF_20)) |
| *Strauchbufo raddei* | still water | 67 | 1575 | NO | YES | 1 | 1 | 20 | ([Kuzmin & Ischenko 1997](#_ENREF_52); [Stöck *et al.* 2000](#_ENREF_92)) |
| **Ranidae** |  |  |  |  |  |  |  |  |  |
| *Abavorana luctuosa* | still water | 68 | 1500 | YES | YES | 1 | 1 | ----- | ([Kueh *et al.* 2010](#_ENREF_49); [Zainudin *et al.* 2010](#_ENREF_105)) |
| *Amnirana galamensis* | still water | 77.4 | 646 | YES | YES | 2 | 2 | ----- | (https://amphibiaweb.org/search/index.html) |
| *Amnirana nicobariensis* | still water | 46.2 | 2650 | YES | YES | 2 | 2 | ----- | ([Inger 1954](#_ENREF_39); [Jehle & Arak 1998](#_ENREF_44)) |
| *Amolops chunganensis* | flowing water | 39 | 3400 | NO | YES | 1 | 1 | 27.4 | ([Matsui *et al.* 1993](#_ENREF_75); [Liu *et al.* 2000](#_ENREF_64)) |
| *Amolops larutensis* | flowing water | 30 | 4900 | NO | YES | 2 | 1 | 20.5 | ([Van Kampen 1923](#_ENREF_102); [Matsui *et al.* 1993](#_ENREF_75)) |
| *Amolops torrentis* | flowing water | 39 | 4318 | YES | YES | 1 | 1 | 23.5 | ([Zhao *et al.* 2017](#_ENREF_108)) |
| *Amolops vitreus* | flowing water | 41.7 | 3155 | NO | YES | 1 | 1 | 17 | ([Le *et al.* 2015](#_ENREF_55)) |
| *Amolops wuyiensis* | flowing water | 47 | 2232 | NO | NO | 2 | 1 | 17.3 | ([Zhang *et al.* 2013](#_ENREF_106)) |
| *Babina adenopleura* | still water | 58 | 1168 | YES | YES | 2 | 1 | 22.5 | ([Fei *et al.* 2012](#_ENREF_30); [Cui *et al.* 2016](#_ENREF_25)) |
| *Babina daunchina* | still water | 51 | 1088 | YES | YES | 1 | 1 | 22.5 | ([Fei *et al.* 2012](#_ENREF_30); [Cui *et al.* 2016](#_ENREF_25)) |
| *Babina hainanensis* | still water | 34 | 772 | YES | YES | 1 | 1 | 22.5 | ([Fei *et al.* 2012](#_ENREF_30); [Cui *et al.* 2016](#_ENREF_25)) |
| *Babina lini* | still water | 61 | 2044 | YES | YES | 1 | 1 | 22.5 | ([Fei *et al.* 2012](#_ENREF_30); [Cui *et al.* 2016](#_ENREF_25)) |
| *Babina pleuraden* | still water | 57 | 469 | YES | YES | 1 | 1 | ----- | ([Fei *et al.* 2012](#_ENREF_30)) (Y. Gong, unpublished data) |
| *Babina subaspera* | still water | 126 | 730 | YES | YES | 1 | 1 | 23 | ([Matsui & Utsunomiya 1983](#_ENREF_74)) (https://amphibiaweb.org/search/index.html) |
| *Clinotarsus curtipes* | still water | 51.4 | 1222 | YES | YES | 2 | 1 | 20.5 | ([Krishna & Krishna 2005](#_ENREF_47)) |
| *Huia cavitympanum* | flowing water | 52 | 15477 | YES | YES | highly variable | highly variable | 25.3 | ([Inger & Stuebing 2005](#_ENREF_42); [Arch *et al.* 2008](#_ENREF_4)) |
| *Huia masonii* | flowing water | 37.8 | 12000 | YES | YES | highly variable | highly variable ----- | | ([Stuart & Chan-Ard 2005](#_ENREF_96); [Boonman & Kurniati 2011](#_ENREF_11)) |
| *Huia sumatrana* | flowing water | ----- | 7594 | YES | YES | 1 | 1 | 23.5 | ([Kurniati 2012](#_ENREF_50)) |
| *Hylarana erythraea* | still water | 48 | 3050 | NO | NO | 1 | 1 | 26.5 | ([Brown & Alcala 1970](#_ENREF_14); [Matsui 1982b](#_ENREF_69); [Roy *et al.* 1995](#_ENREF_85)) |
| *Hylarana guentheri* | still water | 68 | 354 | YES | YES | 1 | 1 | ----- | ([Zhou *et al.* 2014](#_ENREF_109)) (https://amphibiaweb.org/search/index.html) |
| *Hylarana taipehensis* | still water | 30 | 2500 | YES | YES | highly variable | highly variable | 24 | ([Sun & Narins 2005](#_ENREF_100); [Fei *et al.* 2012](#_ENREF_30)) (https://amphibiaweb.org/search/index.html) |
| *Indosylvirana sreeni* | flowing water | 69 | 1837 | NO | NO | 3 | 3 | 20.8 | ([Ganesh & Arumugam 2015](#_ENREF_33)) (https://amphibiaweb.org/search/index.html) |
| *Indosylvirana temporalis* | flowing water | 55.3 | 3100 | NO | NO | 1 | 1 | ----- | ([Kadadevaru *et al.* 2000](#_ENREF_46); [Hampson & Bennett 2002](#_ENREF_37)) |
| *Meristogenys dyscritus* | flowing water | 39.5 | 8000 | YES | YES | 1 | 1 | 17.5 | ([Shimada *et al.* 2011](#_ENREF_91)) |
| *Meristogenys jerboa* | flowing water | 44 | 5650 | YES | YES | 1 | 1 | 25.2 | ([Inger & Gritis 1983](#_ENREF_41); [Matsui *et al.* 1993](#_ENREF_75)) |
| *Meristogenys orphnocnemis* | flowing water | 37.3 | 7205 | NO | NO | 1 | 1 | 24.8 | ([Matsui 1986](#_ENREF_70); [Preininger *et al.* 2007](#_ENREF_82)) |
| *Micrixalus saxicola* | flowing water | 30 | 4771 | NO | NO | 1 | 1 | ----- | ([Krishna & Krishna 2006](#_ENREF_48); [Preininger *et al.* 2013](#_ENREF_83)) |
| *Odorrana amamiensis* | flowing water | 69 | 2165 | YES | YES | 2 | 2 | 15 | ([Matsui 1994](#_ENREF_71)) |
| *Odorrana hosii* | flowing water | 68 | 4950 | YES | YES | highly variable | highly variable ----- | | ([Manthey & Grossmann 1997](#_ENREF_67); [Kurniati *et al.* 2010](#_ENREF_51)) |
| *Odorrana livida* | flowing water | 51 | 15000 | YES | YES | highly variable | highly variable | 22 | ([Ao *et al.* 2003](#_ENREF_2); [Shen *et al.* 2011](#_ENREF_90)) |
| *Odorrana schmackeri* | flowing water | 46 | 3100 | YES | YES | highly variable | 4 | 23 | ([Liu 1950](#_ENREF_63); [Zhang *et al.* 2015](#_ENREF_107)) |
| *Odorrana supranarina* | still water | 76.8 | 1840 | YES | YES | 1 | 1 | 15.2 | ([Matsui 1994](#_ENREF_71)) (https://amphibiaweb.org/search/index.html) |
| *Odorrana tormota* | flowing water | 36 | 19000 | YES | YES | highly variable | highly variable | 17.5 | ([Narins *et al.* 2004](#_ENREF_78); [Fei *et al.* 2012](#_ENREF_30)) |
| *Odorrana utsunomiyaorum* | flowing water | 48.1 | 2326 | YES | YES | 1 | 1 | 15.4 | ([Matsui 1994](#_ENREF_71)) |
| *Pelophylax epeiroticus* | still water | ----- | ----- | YES | NO | 1 | 1 | 26.5 | ([Plötner *et al.* 2010](#_ENREF_80)) (https://amphibiaweb.org/search/index.html) |
| *Pelophylax nigromaculatus* | still water | 70 | 1873 | NO | NO | 1 | 1 | ----- | ([Fei *et al.* 2012](#_ENREF_30)) (L. Zhao, unpublished data) |
| *Pulchrana baramica* | still water | 55.6 | 2289 | YES | YES | 2 | 2 | 25.6 | ([Leong *et al.* 2003](#_ENREF_58)) (https://amphibiaweb.org/search/index.html) |
| *Pulchrana glandulosa* | still water | 55 | 1200 | YES | YES | 1 | 1 | ----- | ([Brown 1902](#_ENREF_12); [Zainudin *et al.* 2010](#_ENREF_105)) |
| *Pulchrana laterimaculata* | still water | 39 | 3245 | YES | YES | 2 | 2 | 25.6 | ([Leong *et al.* 2003](#_ENREF_58)) |
| *Rana cascadae* | still water | 55 | 1309 | YES | YES | 1 | 1 | 25 | (https://amphibiaweb.org/search/index.html) (http://www.westernsoundscape.org/) |
| *Rana dalmatina* | still water | 59 | 682 | NO | NO | 1 | 1 | ----- | ([Lesbarrères & Lodé 2002](#_ENREF_59); [Hettyey *et al.* 2005](#_ENREF_38)) (https://amphibiaweb.org/search/index.html) |
| *Rana dybowskii* | still water | 74.2 | 1376 | NO | YES | 1 | 1 | 9 | ([Cheong & Yang 2000](#_ENREF_18); [Matsui 2014](#_ENREF_72)) (https://amphibiaweb.org/search/index.html) |
| *Rana forreri* | still water | 90 | 850 | NO | NO | 1 | 1 | 21 | ([Frost 1982](#_ENREF_32); [Savage 2002](#_ENREF_88)) |
| *Rana kukunoris* | still water | 62 | 1242 | YES | YES | 1 | 1 | 10.7 | ([Fei *et al.* 2012](#_ENREF_30)) (Y. Cai, unpublished data) |
| *Rana luteiventris* | still water | 71 | 463 | NO | NO | 1 | 1 | 15 | ([Davis & Verrell 2005](#_ENREF_27)) (https://amphibiaweb.org/search/index.html) (http://www.westernsoundscape.org/) |
| *Rana okaloosae* | flowing water | 53.9 | 1273 | NO | NO | 2 | 2 | 25 | ([Bishop 2005](#_ENREF_7)) |
| *Rana palustris* | still water | 56.5 | 1264 | NO | NO | 1 | 1 | 15 | ([Feinberg *et al.* 2014](#_ENREF_31)) (https://amphibiaweb.org/search/index.html) |
| *Rana pretiosa* | still water | 75 | 829 | NO | NO | 1 | 1 | ----- | ([Cushman & Pearl 2007](#_ENREF_26)) (https://amphibiaweb.org/search/index.html) |
| *Rana pyrenaica* | flowing water | 46 | 926 | NO | NO | 1 | 1 | ----- | (https://amphibiaweb.org/search/index.html) |
| *Rana septentrionalis* | still water | 63.1 | 605 | YES | YES | 2 | 2 | 16.1 | ([Leclair & Laurin 1996](#_ENREF_56); [Bevier *et al.* 2004](#_ENREF_6)) |
| *Rana sphenocephala* | still water | 84.1 | 1215 | YES | YES | 1 | 1 | 11 | ([Chu & Wilczynski 2001](#_ENREF_21); [Feinberg *et al.* 2014](#_ENREF_31)) (https://amphibiaweb.org/search/index.html) |
| *Rana sylvatica* | still water | ----- | 1427 | YES | YES | 2 | 1 | 10.1 | ([Feinberg *et al.* 2014](#_ENREF_31)) (https://amphibiaweb.org/search/index.html) |
| *Rana uenoi* | still water | 62.3 | 1220 | YES | YES | 1 | 1 | 4.7 | ([Matsui 2014](#_ENREF_72)) |
| *Rana vaillanti* | still water | 94 | 2562 | YES | YES | highly variable | highly variable ----- | | (https://amphibiaweb.org/search/index.html) |
| *Sanguirana acai* | flowing water | 57.6 | 1650 | NO | NO | 1 | 1 | 24 | ([Brown *et al.* 2017](#_ENREF_13)) |
| *Staurois guttatus* | flowing water | 33.4 | 4195 | YES | YES | 1 | 1 | 25.5 | ([Preininger *et al.* 2016](#_ENREF_84)) |
| *Staurois latopalmatus* | flowing water | 47.7 | 5165 | NO | NO | 1 | 1 | 26.4 | ([Boeckle *et al.* 2009](#_ENREF_8)) |
| *Staurois parvus* | flowing water | 23.6 | 5578 | NO | NO | 1 | 1 | 25.5 | ([Matsui *et al.* 2007](#_ENREF_73); [Grafe *et al.* 2012](#_ENREF_35)) |
| **Note:**  1. All data from https://amphibiaweb.org/index.html were cited as follow: AmphibiaWeb. 2019. <http://amphibiaweb.org> University of California, Berkeley, CA, USA. Accessed 18 Mar 2019. 2. Red represents the data of frogs that come from our own measurements of recordings. | | | | | | | | | |

**Reference**

Alonso, R. & Rodríguez, A. (2003). Advertisement calls of Cuban toads of the genus Bufo (Anura, Bufonidae). *Phyllomedusa*, 2, 75-82.

Ao, J.M., Bordoloi, S. & Ohler, A. (2003). Amphibian fauna of Nagaland with nineteen new records from the state including five new records for India. *Zoos' Print Journal*, 18, 1117-1125.

Arak, A. (1988). Female mate selection in the natterjack toad: active choice or passive attraction? *Behavioral Ecology and Sociobiology*, 22, 317-327.

Arch, V.S., Grafe, T.U. & Narins, P.M. (2008). Ultrasonic signalling by a Bornean frog. *Biology Letters*, 4, 19-22.

Arch, V.S., Richards-Zawaki, C.L. & Feng, A.S. (2011). Acoustic communication in the Kihansi Spray Toad (*Nectophrynoides asperginis*): insights from a captive population. *Journal of Herpetology*, 45, 45-49.

Bevier, C.R., Larson, K., Reilly, K. & Tat, S. (2004). Vocal repertoire and calling activity of the mink frog, *Rana septentrionalis*. *Amphibia-Reptilia*, 25, 255-264.

Bishop, D.C. (2005). *Ecology and distribution of the Florida bog frog and flatwoods salamander on Eglin Air Force Base*. Diss. Virginia Tech.

Boeckle, M., Preininger, D. & Hödl, W. (2009). Communication in noisy environments I: acoustic signals of Staurois latopalmatus Boulenger 1887. *Herpetologica*, 65, 154-165.

Bogert, C.M. (1962). Isolation mechanisms in toads of the *Bufo debilis* group in Arizona and western Mexico. *American Museum Novitates*, 2100, 1-37.

Boistel, R., Aubin, T., Cloetens, P., Langer, M., Gillet, B., Josset, P. *et al.* (2011). Whispering to the deaf: communication by a frog without external vocal sac or tympanum in noisy environments. *PLoS One*, 6, e22080.

Boonman, A. & Kurniati, H. (2011). Evolution of high-frequency communication in frogs. *Evolutionary Ecology Research*, 13, 197–207.

Brown, A.E. (1902). A collection of reptiles and batrachians from Borneo and the Loo Choo Islands. *Proceedings of the Academy of Natural Sciences of Philadelphia*, 54, 175-186.

Brown, R.M., Prue, A., Onn, C.K., Gaulke, M., Sanguila, M.B. & Siler, C.D. (2017). Taxonomic reappraisal of the northeast Mindanao Stream Frog, *Sanguirana albotuberculata* (Inger 1954), validation of *Rana mearnsi*, Stejneger 1905, and description of a new species from the central Philippines. *Herpetological Monographs*, 31, 210-231.

Brown, W.C. & Alcala, A.C. (1970). Population ecology of the frog *Rana erythraea* in southern Negros, Philippines. *Copeia*, 1970, 611-622.

Caldwell, J.P. & Shepard, D.B. (2007). Calling site fidelity and call structure of a Neotropical toad, *Rhinella ocellata* (Anura: Bufonidae). *Journal of Herpetology*, 41, 611-621.

Castellano, S., Rosso, A., Doglio, S. & Giacoma, C. (1999). Body size and calling variation in the green toad (*Bufo viridis*). *Journal of Zoology*, 248, 83-90.

Chandramouli, S.R., Vasudevan, K., Harikrishnan, S., Dutta, S.K., Janani, S.J., Sharma, R. *et al.* (2016). A new genus and species of arboreal toad with phytotelmonous larvae, from the Andaman Islands, India (Lissamphibia, Anura, Bufonidae). *ZooKeys*, 57-90.

Cheong, P.S.R.S. & Yang, S.Y. (2000). Call types of dybowski's brown frog and their variations from two recording areas. *The Korean Journal of Ecology*, 23, 309-313.

Cherry, M.I. & Francillon-Vieillot, H. (1992). Body size, age and reproduction in the leopard toad, *Bufo pardalis*. *Journal of Zoology*, 228, 41-50.

Cherry, M.I. & Grant, W.S. (1994). Phylogenetic relationships and call structure in four African bufonid species. *South African Journal of Zoology*, 29, 1-10.

Chu, J. & Wilczynski, W. (2001). Social influences on androgen levels in the southern leopard frog, *Rana sphenocephala*. *General and Comparative Endocrinology*, 121, 66-73.

Cocroft, R.B., McDiarmid, R.W., Jaslow, A.P. & Ruiz-Carranza, P.M. (1990). Vocalizations of eight species of Atelopus (Anura: Bufonidae) with comments on communication in the genus. *Copeia*, 1990, 631-643.

Cocroft, R.B. & Ryan, M.J. (1995). Patterns of advertisement call evolution in toads and chorus frogs. *Animal Behaviour*, 49, 283-303.

Coloma, L.A., Lötters, S. & Salas, A.W. (2000). Taxonomy of the Atelopus ignescens complex (Anura: Bufonidae): designation of a neotype of Atelopus ignescens and recognition of Atelopus exiguus. *Herpetologica*, 56, 303-324.

Cui, J., Wang, J., Fang, G., Song, X., Brauth, S.E. & Tang, Y. (2016). Coevolution of male and female response preferences to sexual signals in music frogs. *Asian Herpetological Research*, 7, 87-95.

Cushman, K.A. & Pearl, C.A. (2007). A conservation assessment for the Oregon spotted frog (Rana pretiosa), USDA Forest Service and USDI Bureau of Land Management. *Oregon*.

Davis, A.B. & Verrell, P.A. (2005). Demography and reproductive ecology of the Columbia spotted frog (*Rana luteiventris*) across the Palouse. *Canadian Journal of Zoology*, 83, 702-711.

de Carvalho, T.R., de Magalhães Tolentino, V.C. & Giaretta, A.A. (2013). Advertisement call of *Rhinella pygmaea* (Myers and Carvalho, 1952) (Anura: Bufonidae) from the northern State of Rio de Janeiro. *Herpetology Notes*, 6, 229-231.

Diego-Rasilla, F.J. & Luengo, R.M. (2004). Heterospecific call recognition and phonotaxis in the orientation behavior of the marbled newt, *Triturus marmoratus*. *Behavioral Ecology and Sociobiology*, 55, 556-560.

Fei, L., Ye, C.Y. & Jiang, J.P. (2012). *Colored atlas of Chinese amphibians and their distributions*. Sichuan Publishing House of Science & Technology, Chengdu.

Feinberg, J.A., Newman, C.E., Watkins-Colwell, G.J., Schlesinger, M.D., Zarate, B., Curry, B.R. *et al.* (2014). Cryptic diversity in metropolis: confirmation of a new leopard frog species (Anura: Ranidae) from New York City and surrounding Atlantic coast regions. *PLoS One*, 9, e108213.

Frost, J.S. (1982). Functional genetic similarity between geographically separated populations of Mexican Leopard Frogs (*Rana pipiens* complex). *Systematic Zoology*, 31, 57-67.

Ganesh, S.R. & Arumugam, M. (2015). Natural History and distribution notes on the Sreeni's golden frog (*Indosylvirana sreeni*) in the Southern Eastern Ghats, peninsular India. *Alytes*, 32, 59-65.

Giacoma, C., Zugolaro, C. & Beani, L. (1997). The advertisement calls of the green toad (*Bufo viridis*): variability and role in mate choice. *Herpetologica*, 53, 454-464.

Grafe, T.U., Preininger, D., Sztatecsny, M., Kasah, R., Dehling, J.M., Proksch, S. *et al.* (2012). Multimodal communication in a noisy environment: a case study of the Bornean rock frog *Staurois parvus*. *PLoS One*, 7, e37965.

Hödl, W. (1990). Reproductive diversity in Amazonian lowland frogs. *Fortschritte der Zoologie*, 38, 41-60.

Hampson, K. & Bennett, D. (2002). Advertisement calls of amphibians at Lackunda Estate, Coorg, Karnataka. In: *Frogs of Coorg, Karnataka, India, Vol. 11* (ed. Bennett, D). Viper Press, pp. 121-135.

Hettyey, A., Török, J., Hévizi, G. & Fox, S. (2005). Male mate choice lacking in the agile frog, *Rana dalmatina*. *Copeia*, 2005, 403-408.

Inger, R.F. (1954). Systematics and zoogeography of Philippine Amphibia. *Fieldiana Zoology*, 39, 183-531.

Inger, R.F. (1964). Two new species of frogs from Borneo. *Fieldiana Zoology*, 44, 151-159.

Inger, R.F. & Gritis, P.A. (1983). Variation in Bornean frogs of the *Amolops jerboa* species group, with description of two new species. *Fieldiana Zoology*, 19, 1-13.

Inger, R.F. & Stuebing, R.B. (2005). A field guide to the frogs of Borneo, Kota Kinabalu, Borneo.

Jaslow, A.P. (1979). Vocalization and aggression in *Atelopus chiriquiensis* (Amphibia, Anura, Bufonidae). *Journal of Herpetology*, 13, 141-145.

Jehle, R. & Arak, A. (1998). Graded call variation in the Asian cricket frog *Rana nicobariensis*. *Bioacoustics*, 9, 35-48.

Köhler, J. & Lötters, S. (1999). Annotated list of amphibian records from the Departamento Pando, Bolivia, with description of some advertisement calls. *Bonner Zoologische Beiträege*, 48, 259-273.

Kadadevaru, G.G., Kanamadi, R.D. & Schneider, H. (2000). Advertisement call of the Indian Bronzed Frog, *Rana temporalis* (Gunther, 1864). *Herpetological Bulletin*, 73, 8-9.

Krishna, S.N. & Krishna, S.B. (2005). Female courtship calls of the litter frog (*Rana curtipes*) in the tropical forests of Western Ghats, South India. *Amphibia-Reptilia*, 26, 431-435.

Krishna, S.N. & Krishna, S.B. (2006). Visual and acoustic communication in an endemic stream frog, *Micrixalus saxicolus* in theWestern Ghats, India. *Amphibia-Reptilia*, 143-147.

Kueh, B.H., Ismail, N., Lau, C.E.S., Albert, J., Siwan, E.S. & Ngidang, V.B.A. (2010). *Rana Luctuosa* (Mahogany Frog). Altitude and maximum size. *Herpetological Review*, 41, 341-342.

Kurniati, H. (2012). Ecological assessment of sumatran torrent frogs.

Kurniati, H., Sumadijaya, A., Boonman, A. & Laksono, W.T. (2010). Ecology, distribution and bio-acoustic of amphibians in degraded habitat. *Indonesian Institute of Science, Cibinong*.

Kuzmin, S.L. & Ischenko, V.G. (1997). Skeletochronology of *Bufo raddei* from the Gobi Desert. *Journal of Herpetology*, 31, 306-309.

Lötters, S., Glaw, F., Reichle, S., Köhler, J. & Meyer, E. (1999). Notes on vocalizations in three species of Atelopus from Central and South America. *Herpetozoa*, 12, 79-83.

Lötters, S., Haas, W., Schick, S. & Böhme, W. (2002). On the systematics of the harlequin frogs (Amphibia: Bufonidae: Atelopus) from Amazonia. II: Redescription of *Atelopus pulcher* (BOULENGER, 1882) from the eastern Andean versant in Peru. *Salamandra*, 38, 164-184.

Le, D.T., Pham, A.V., Nguyen, S.H.L., Ziegler, T. & Nguyen, T.Q. (2015). First records of *Megophrys daweimontis* Rao and Yang, 1997 and *Amolops vitreus* (Bain, Stuart and Orlov, 2006)(Anura: Megophryidae, Ranidae) from Vietnam. *Asian Herpetological Research*, 6, 66-72.

Leclair, R. & Laurin, G. (1996). Growth and body size in populations of mink frogs *Rana septentrionalis* from two latitudes. *Ecography*, 19, 296-304.

Lee, Y.H. & Stuebing, R.B. (1990). Heavy metal contamination in the River Toad,*Bufo juxtasper* (Inger), near a copper mine in East Malaysia. *Bulletin of Environmental Contamination and Toxicology*, 45, 272-279.

Leong, T.M., Matsui, M., Yong, H.S. & HAMID, A.A. (2003). Revalidation of *Rana laterimaculata* Barbour et Noble, 1916 from the synonymy of *Rana baramica* Boettger, 1901. *Current herpetology*, 22, 17-27.

Lesbarrères, D. & Lodé, T. (2002). Variations in male calls and responses to an unfamiliar advertisement call in a territorial breeding anuran,*Rana dalmatina*: evidence for a “dear enemy” effect. *Ethology Ecology & Evolution*, 14, 287-295.

Lescure, J. & Marty, C. (2000). *Atlas des amphibiens de Guyane*. Publications Scientifiques du M.N.H.N., Paris.

Lima, A.P., Magnusson, W.E., Menin, M., Erdtmann, L.K., Rodrigues, D.J., Keller, C. *et al.* (2006). Guia de Sapos da Reserva Adolpho Ducke: Amazônia Central Áttema Design Editorial, Manaus.

Lima, A.P., Menin, M. & Araújo, M.C. (2007). A new species of Rhinella (Anura: Bufonidae) from Brazilian Amazon. *Zootaxa*, 1663, 1-15.

Liu, C.C. (1950). *Amphibians of Western China*. Chicago Natural History Museum, Chicago.

Liu, W., Yang, D., Ferraris, C. & Matsui, M. (2000). *Amolops bellulus*: A new species of stream-breeding frog from western Yunnan, China (Anura: Ranidae). *Copeia*, 2000, 536-541.

Lo Valvo, M. & Giacalone, G. (2013). Biometrical analyses of a Sicilian green toad, *Bufo siculus* (Stöck et al. 2008), population living in Sicily (Italy). *International Journal of Morphology*, 31, 681-686.

Malkmus, R., Manthey, U., Vogel, G., Hoffmann, P. & Kosuch, J. (2002). *Amphibians & reptiles of Mount Kinabalu (North Borneo)*. Koeltz Scientific Books, Berlin.

Manthey, U. & Grossmann, W. (1997). *Amphibien und reptilien südostasiens*. Natur und Tier Verlag, Münster, Germany.

Matsui, M. (1982a). Call characteristics of several anuran species from East Kalimantan. *Contributions from the Biological Laboratory, Kyoto University*, 26, 131-139.

Matsui, M. (1982b). Amphibians from Sabah II. Acoustic characteristics of three common anuran species. *Contributions from the Biological Laboratory, Kyoto University*, 26, 123-129.

Matsui, M. (1986). Three new species of Amolops from Borneo (Amphibia, Anura, Ranidae). *Copeia*, 1986, 623-630.

Matsui, M. (1994). A taxonomic study of the *Rana narina* complex, with description of three new species (Amphibia: Ranidae). *Zoological Journal of the Linnean Society*, 111, 385-415.

Matsui, M. (2014). Description of a new brown frog from Tsushima Island, Japan (Anura: Ranidae: Rana). *Zoological Science*, 31, 613-620.

Matsui, M., Mohamed, M., Shimada, T. & Sudin, A. (2007). Resurrection of *Staurois parvus* from S. tuberilinguis from Borneo (Amphibia, Ranidae). *Zoological Science*, 24, 101-106.

Matsui, M. & Utsunomiya, T. (1983). Mating call characteristics of the frogs of the subgenus Babina with reference to their relationship with *Rana adenopleura*. *Journal of Herpetology*, 17, 32-37.

Matsui, M., Wu, G.F. & Yong, H.S. (1993). Acoustitic characteristics of three species of the genus Amolops (Amphibia, Anura, Ranidae). *Zoological Science*, 10, 691-695.

McDiarmid, R.W. & Foster, M.S. (1981). Breeding habits of the toad *Bufo coccifer* in Costa Rica, with a description of the tadpole. *Southwestern Naturalist*, 26, 353-363.

Mendelson III, J.R., Williams, B.L., Sheil, C.A. & Mulcahy, D.G. (2005). Systematics of the *Bufo coccifer* complex (Anura: Bufonidae) of Mesoamerica. *Scientific Papers Natural History Museum, The University of Kansas*, 38, 1-27.

Narins, P.M., Feng, A.S., Lin, W., Schnitzler, H.U., Denzinger, A., Suthers, R.A. *et al.* (2004). Old world frog and bird vocalizations contain prominent ultrasonic harmonics. *The Journal of the Acoustical Society of America*, 115, 910-913.

Narvaes, P. & Rodrigues, M.T. (2009). Taxonomic revision of *Rhinella granulosa* species group (Amphibia, Anura, Bufonidae), with a description of a new species. *Arquivos de Zoologia*, 40, 1-73.

Plötner, J., Uzzell, T., Beerli, P., Akın, C., Bilgin, C.C., Haefeli, C. *et al.* (2010). Genetic divergence and evolution of reproductive isolation in eastern Mediterranean water frogs. In: *Evolution in action*. Springer, Berlin, Heidelberg, pp. 373-403.

Porter, K.R. (1966). Mating calls of six Mexican and Central American toads (genus Bufo). *Herpetologica*, 22, 60-67.

Preininger, D., Böeckle, M. & Hödl, W. (2007). Comparison of anuran acoustic communities of two habitat types in the Danum Valley Conservation Area, Sabah, Malaysia. *Salamandra*, 43, 129-138.

Preininger, D., Boeckle, M., Freudmann, A., Starnberger, I., Sztatecsny, M. & Hodl, W. (2013). Multimodal signaling in the Small Torrent Frog (*Micrixalus saxicola*) in a complex acoustic environment. *Behavioral Ecology and Sociobiology*, 67, 1449-1456.

Preininger, D., Handschuh, S., Boeckle, M., Sztatecsny, M. & Hödl, W. (2016). Comparison of female and male vocalisation and larynx morphology in the size dimorphic foot-flagging frog species *Staurois guttatus*. *Herpetological Journal*, 26, 187-197.

Roy, D., Borah, B. & Sarma, A. (1995). Analysis and significance of female reciprocal call in frogs. *Current Science*, 69, 265-270.

Salas, N.E., Zavattieri, M.V., Di Tada, I.E., Martino, A.L. & Bridarolli, M.E. (1998). Bioacoustical and etho-ecological features in amphibian communities of southern Cordoba Province (Argentina). *Cuadernos de Herpetolgia*, 12, 37-46.

Sao-Pedro, V.A., Medeiros, P.H. & Garda, A.A. (2011). The advertisement call of *Rhinella granulosa* (Anura, Bufonidae). *Zootaxa*, 3092, 60-62.

Savage, J.M. (2002). *The amphibians and reptiles of Costa Rica: a herpetofauna between two continents, between two seas*. University of Chicago Press.

Schwartz, A. (1972). The native toads (Anura, Bufonidae) of Hispaniola. *Journal of Herpetology*, 6, 217-231.

Shen, J.X., Xu, Z.M., Feng, A.S. & Narins, P.M. (2011). Large odorous frogs (*Odorrana graminea*) produce ultrasonic calls. *Journal of Comparative Physiology A*, 197, 1027-1030.

Shimada, T., Matsui, M., Yambun, P. & Sudin, A. (2011). A survey of morphological variation in adult *Meristogenys amoropalamus* (Amphibia, Anura, Ranidae), with a description of a new cryptic species. *Zootaxa*, 2905, 33-56.

Stöck, M., Bretschneider, P. & Grosse, W. (2000). The mating call and male release call of *Bufo raddei* Strauch, 1876 with some phylogenetic implications. *Russian Journal of Herpetology*, 7, 215-226.

Stöck, M., Sicilia, A., Belfiore, N.M., Buckley, D., Lo Brutto, S., Lo Valvo, M. *et al.* (2008). Post-Messinian evolutionary relationships across the Sicilian channel: mitochondrial and nuclear markers link a new green toad from Sicily to African relatives. *BMC Evolutionary Biology*, 8, 56.

Starnberger, I., Kamminga, P., Fosah, V.C. & Nuttman, C. (2011). The 'push-up'as a calling posture in *Nectophrynoides tornieri* (Anura: Bufonidae) in the Amani Nature Reserve, Tanzania. *Herpetologica*, 67, 124-134.

Straneck, R., Olmedo, E.V. & Carrizo, G.R. (1993). *Catalogo de voces de anfíbios Argentinos. Parte 1*. Ediciones Lola, Buenos Aires.

Stuart, B.L. & Chan-Ard, T. (2005). Two new Huia (Amphibia: Ranidae) from Laos and Thailand. *Copeia*, 2005, 279-289.

Suazo-Ortuño, I., Alvarado-Díaz, J., Raya-Lemus, E. & Martinez-Ramos, M. (2007). Diet of the Mexican marbled toad (*Bufo marmoreus*) in conserved and disturbed tropical dry forest. *Southwestern Naturalist*, 52, 305-309.

Sullivan, B.K. (1983). Sexual selection in the great plains toad (*Bufo cognatus*). *Behaviour*, 258-264.

Sullivan, B.K. & Wagner Jr, W.E. (1988). Variation in advertisement and release calls, and social influences on calling behavior in the Gulf Coast toad (*Bufo valliceps*). *Copeia*, 1988, 1014-1020.

Sun, J.W.C. & Narins, P.M. (2005). Anthropogenic sounds differentially affect amphibian call rate. *Biological Conservation*, 121, 419-427.

Sun, Z.X. (2017). *A comparison of acoustic structure of vocalization in different habitat frog species in the Mt. Diaoluo National Nature Reserve*. Hainan Normal University, Haikou.

Van Kampen, P.N. (1923). The Amphibia of the Indo-Australian Archipelago E. J. Brill, Leiden.

Wagner Jr, W.E. & Sullivan, B.K. (1995). Sexual selection in the Gulf Coast toad, *Bufo valliceps*-female choice based on variable characters. *Animal Behaviour*, 49, 305-319.

Wood Jr., P.L., Grismer, L.L., Ahmad, N. & Senawi, J. (2008). Two new species of torrent-dwelling toads *Ansonia Stoliczka*, 1870 (Anura: Bufonidae) from Peninsular Malaysia. *Herpetologica*, 64, 321-340.

Zainudin, R., Rahman, M.A., Zain, B.M.M., Shukor, M.N., Inger, R.F. & Norhayati, A. (2010). Mating calls description of five species of frogs from the genus *Hylarana* Tschudi 1838 (Amphibia, Anura, Ranidae) from Sarawak, Malaysia. *Sains Malaysiana*, 39, 363-369.

Zhang, F., Chen, P. & Zhao, S. (2013). Comparison of mating calls and adaptive strategies of *Amolops wuyiensis* and *Odorrana tormotus* (Anura) in noise-controlled environments. *Zoological Research*, 34, 196-203.

Zhang, F., Chen, Z., Zhao, J. & Chen, P. (2015). Analyzing the characteristics of courtship calls of *Odorrana schmackeri* in noisy environment. *Chinese Journal of Zoology*, 50, 52-58.

Zhao, L., Wang, J., Yang, Y., Zhu, B., Brauth, S.E., Tang, Y. *et al.* (2017). An exception to the matched filter hypothesis: A mismatch of male call frequency and female best hearing frequency in a torrent frog. *Ecology and Evolution*, 7, 419-428.

Zhou, Y.L., Qiu, X., Fang, X.B., Yang, L.Y., Zhao, Y., Fang, T. *et al.* (2014). Acoustic characteristics of eight common Chinese anurans during the breeding season. *Dong wu xue yan jiu = Zoological research*, 35, 42-50.

Zweifel, R.G. (1968). Effects of temperature, body size, and hybridization on mating calls of toads, *Bufo a. americanus* and *Bufo woodhousii fowleri*. *Copeia*, 1968, 269-285.
