# Supplementary material for: Noise constrains the evolution of call frequency contours in flowing water frogs: a comparative analysis in two clades
Source: Front Zool. 2021 Aug 4;18:37. doi: 10.1186/s12983-021-00423-y (PMC8336270; doi:10.1186/s12983-021-00423-y)
Supplement: Supplementary file 2 — Additional file 2: Table S2. Name, sound pressure levels (SPLs), habitat types and data sources for all species. [file 12983_2021_423_MOESM2_ESM.docx]

| **Taxa** | **SPL** | **Habitat type** | **Sources** |
| --- | --- | --- | --- |
| *Amolops chunganensis F11* | 61.44 | Flowing water | Goutte et al. 2018 |
| *Amolops chunganensis S9* | 48.81 | Flowing water | Goutte et al. 2018 |
| *Amolops torrentis* | 63.50 | Flowing water | Zhao et al. 2017 |
| *Amolops wuyiensis* | 65.20 | Flowing water | Zhang et al. 2013 |
| *Atelopus francisus* | 57.09 | Flowing water | Boistel et al. 2011 |
| *Huia cavitympanum* | 70.52 | Flowing water | Goutte et al. 2018 |
| *Meristogenys amoropalamus* | 60.99 | Flowing water | Goutte et al. 2018 |
| *Meristogenys kinabaluensis* | 67.83 | Flowing water | Goutte et al. 2018 |
| *Meristogenys spa* | 70.53 | Flowing water | Goutte et al. 2018 |
| *Meristogenys spb* | 73.74 | Flowing water | Goutte et al. 2018 |
| *Micrixalus saxicola* | 67.00 | Flowing water | Preininger et al. 2013 |
| *Odorrana graminea F3* | 64.22 | Flowing water | Goutte et al. 2018 |
| *Odorrana graminea S20* | 63.40 | Flowing water | Goutte et al. 2018 |
| *Odorrana hosii SG49* | 74.26 | Flowing water | Goutte et al. 2018 |
| *Odorrana tormotus* | 65.20 | Flowing water | Zhang et al. 2013 |
| *Odorrana yizhangensis* | 62.04 | Flowing water | Goutte et al. 2018 |
| *Rhacophorus gauni* | 72.90 | Flowing water | Goutte et al. 2018 |
| *Staurois guttatus* | 78.22 | Flowing water | Goutte et al. 2018 |
| *Staurois latopalmatus* | 81.80 | Flowing water | Boeckle et al. 2009 |
| *Staurois parvus* | 72.00 | Flowing water | Grafe et al. 2012 |
| *Staurois tuberilinguis F23* | 74.80 | Flowing water | Goutte et al. 2018 |
| *Staurois tuberilinguis SG61* | 76.15 | Flowing water | Goutte et al. 2018 |
| *Babina adenopleura* | 44.40 | Still water | Goutte et al. 2018 |
| *Babina daunchina* | 43.80 | Still water | Goutte et al. 2018 |
| *Fejervarya limnocharis SG18* | 71.50 | Still water | Goutte et al. 2018 |
| *Hyla annectans* | 56.00 | Still water | Goutte et al. 2018 |
| *Hylarana chalconota* | 57.80 | Still water | Goutte et al. 2018 |
| *Hylarana guentheri F13* | 51.63 | Still water | Goutte et al. 2018 |
| *Hylarana mortenseni* | 55.06 | Still water | Goutte et al. 2018 |
| *Hylarana nicobariensis* | 57.84 | Still water | Goutte et al. 2018 |
| *Microhyla berdmorei* | 45.00 | Still water | Goutte et al. 2018 |
| *Microhyla heymonsi* | 56.80 | Still water | Goutte et al. 2018 |
| *Odorrana schmackeri* | 51.05 | Still water | Goutte et al. 2018 |
| *Polypedates leucomystax* | 52.70 | Still water | Goutte et al. 2018 |
| *Polypedates megacephalus F2* | 55.90 | Still water | Goutte et al. 2018 |
| *Rhacophorus dugritei* | 56.30 | Still water | Goutte et al. 2018 |

**References**

Boeckle, M., Preininger, D. & Hödl, W. (2009). Communication in noisy environments I: acoustic signals of *Staurois latopalmatus* Boulenger 1887. *Herpetologica*, 65, 154-165.

Boistel, R., Aubin, T., Cloetens, P., Langer, M., Gillet, B., Josset, P. *et al.* (2011). Whispering to the deaf: communication by a frog without external vocal sac or tympanum in noisy environments. *PLoS One*, 6, e22080.

Goutte, S., Dubois, A., Howard, S.D., Marquez, R., Rowley, J.J.L., Dehling, J.M. *et al.* (2018). How the environment shapes animal signals: a test of the acoustic adaptation hypothesis in frogs. *Journal of Evolutionary Biology*, 31, 148-158.

Grafe, T.U., Preininger, D., Sztatecsny, M., Kasah, R., Dehling, J.M., Proksch, S. *et al.* (2012). Multimodal communication in a noisy environment: a case study of the Bornean rock frog *Staurois parvus*. *PLoS One*, 7, e37965.

Preininger, D., Boeckle, M., Freudmann, A., Starnberger, I., Sztatecsny, M. & Hodl, W. (2013). Multimodal signaling in the Small Torrent Frog (*Micrixalus saxicola*) in a complex acoustic environment. *Behavioral Ecology and Sociobiology*, 67, 1449-1456.

Zhang, F., Chen, P. & Zhao, S. (2013). Comparison of mating calls and adaptive strategies of *Amolops wuyiensis* and *Odorrana tormotus* (Anura) in noise-controlled environments. *Zoological Research*, 34, 196-203.

Zhao, L., Wang, J., Yang, Y., Zhu, B., Brauth, S.E., Tang, Y. *et al.* (2017). An exception to the matched filter hypothesis: A mismatch of male call frequency and female best hearing frequency in a torrent frog. *Ecology and Evolution*, 7, 419-428.
