# Supplementary material for: Noise constrains the evolution of call frequency contours in flowing water frogs: a comparative analysis in two clades
Source: Front Zool. 2021 Aug 4;18:37. doi: 10.1186/s12983-021-00423-y (PMC8336270; doi:10.1186/s12983-021-00423-y)
Supplement: Supplementary file 3 — Additional file 3: Table S3. GenBank accession numbers for all sampled species and outgroup Buergeria buergeri. [file 12983_2021_423_MOESM3_ESM.docx]

**Bufonidae**

| **Species** | **12S** | **16S** | **CXCR4** | **RAG1** | **RHOD** |
| --- | --- | --- | --- | --- | --- |
| *Anaxyrus americanus* | FJ882827 | DQ158426 | DQ306520 | DQ158352 | --- |
| *Anaxyrus boreas* | DQ283180 | AY325983 | DQ306499 | DQ158360 | DQ283871 |
| *Anaxyrus cognatus* | DQ158444 | DQ158444 | DQ306498 | DQ158367 | KJ532267 |
| *Anaxyrus fowleri* | DQ158451 | AY680224 | DQ306505 | DQ158373 | EF372180 |
| *Anaxyrus retiformis* | AY325982 | AY325982 | --- | --- | --- |
| *Ansonia hanitschi* | AB331710 | FJ882794 | FJ882695 | --- | --- |
| *Ansonia leptopus* | FJ882795 | FJ882795 | FJ882697 | --- | --- |
| *Ansonia longidigita* | FJ882796 | FJ882796 | FJ882698 | KF666400 | DQ283968 |
| *Ansonia platysoma* | AB435272 | AB435272 | --- | --- | --- |
| *Atelopus chiriquiensis* | U52735 | U52780 | --- | --- | --- |
| *Atelopus franciscus* | --- | JQ742150 | --- | --- | --- |
| *Atelopus peruensis* | DQ158419 | DQ158419 | DQ306495 | DQ158345 | --- |
| *Atelopus pulcher* | --- | EU672973 | --- | --- | --- |
| *Atelopus spumarius* | DQ283260 | DQ283260 | GU183852 | --- | DQ283929 |
| *Atelopus tricolor* | GU301900 | EU672978 | --- | --- | --- |
| *Atelopus varius* | AY325996 | AY325996 | --- | --- | --- |
| *Atelopus zeteki* | --- | DQ283252 | --- | --- | --- |
| *Bufotes siculus* | --- | EU497446 | --- | EU497609 | --- |
| *Bufotes viridis* | AY680267 | FJ882813 | FJ882714 | EU497603 | DQ283940 |
| *Epidalea calamita* | FJ882809 | FJ882809 | FJ882709 | EU497610 | --- |
| *Incilius coccifer* | DQ158443 | DQ158443 | DQ306526 | DQ158366 | KJ532276 |
| *Incilius coniferus* | DQ158445 | DQ283166 | DQ306534 | DQ158368 | DQ283860 |
| *Incilius ibarrai* | AY680249 | AY680249 | HM563906 | HM563992 | --- |
| *Incilius luetkenii* | DQ158467 | DQ158467 | DQ306565 | DQ158387 | KJ532275 |
| *Incilius marmoreus* | AY680250 | AY680250 | HM563913 | KJ609671 | KJ532278 |
| *Incilius valliceps* | DQ158493 | DQ158493 | DQ306545 | DQ158409 | KJ532273 |
| *Ingerophrynus biporcatus* | AY325987 | AY325987 | KY555668 | KY555715 | --- |
| *Peltophryne cataulaciceps* | --- | JF434577 | JF342406 | JF342364 | --- |
| *Peltophryne empusa* | AF361695 | AY028489 | JF342412 | JF342370 | --- |
| *Peltophryne gundlachi* | AY028479 | AY028492 | JF342423 | JF342381 | --- |
| *Peltophryne longinasus* | AY028480 | AY028493 | JF342426 | JF342384 | --- |
| *Peltophryne taladai* | AY028482 | AY028495 | JF342447 | JF342405 | --- |
| *Rhinella arenarum* | AY843573 | AY843573 | DQ306529 | DQ158354 | AY844547 |
| *Rhinella castaneotica* | DQ158440 | DQ158440 | DQ306539 | DQ158364 | --- |
| *Rhinella fernandezae* | KP685204 | KP685204 | KP684960 | KP685126 | KP685169 |
| *Rhinella granulosa* | FJ882774 | AY028496 | DQ306557 | DQ158380 | DQ283966 |
| *Rhinella ocellata* | DQ158479 | DQ158479 | DQ306538 | DQ158398 | --- |
| *Rhinella pygmaea* | KP685229 | KP685229 | KP684982 | KP685141 | KP685182 |
| *Strauchbufo raddei* | AF160776 | GU183855 | KF666101 | KF666186 | --- |

**Leptodactylidae**

| ***Species*** | **12S** | **16S** | **CXCR4** | **RAG1** | **RHOD** |
| --- | --- | --- | --- | --- | --- |
| *Physalaemus pustulosus* | DQ337242 | DQ337248 | EF107462 | EF107299 | --- |

**Ranidae**

| **Species** | **12S** | **16S** | **CXCR4** | **RAG1** | **RHOD** |
| --- | --- | --- | --- | --- | --- |
| *Abavorana luctuosa* | KF477635 | DQ861315 | --- | KR264407 | --- |
| *Amnirana galamensis* | DQ347032 | DQ283058 | KR264338 | DQ347260 | AY322238 |
| *Amnirana nicobariensis* | AY326062 | AY326062 | --- | DQ347274 | DQ347393 |
| *Babina adenopleura* | DQ359957 | DQ283117 | --- | KU840714 | DQ283829 |
| *Babina daunchina* | KU840524 | KF185065 | --- | KU840723 | --- |
| *Babina hainanensis* | MF807899 | MF807821 | --- | --- | --- |
| *Babina lini* | MF807898 | KF185066 | --- | --- | --- |
| *Babina pleuraden* | DQ359958 | DQ359989 | KR264303 | KR264384 | DQ360011 |
| *Babina subaspera* | NC_022871 | NC_022871 | --- | --- | --- |
| *Clinotarsus curtipes* | DQ346968 | GU136111 | --- | DQ347209 | AF249117 |
| *Huia cavitympanum* | AB211466 | AB211489 | --- | EF088246 | KU840634 |
| *Huia masonii* | DQ347021 | DQ347313 | --- | EF088247 | DQ347372 |
| *Huia sumatrana* | AB211468 | AB211491 | --- | EF088249 | --- |
| *Hylarana erythraea* | AF206094 | AF206475 | KR264318 | EF088268 | AY322228 |
| *Hylarana guentheri* | DQ283266 | AF206476 | KR264284 | KU840700 | DQ360024 |
| *Hylarana taipehensis* | DQ283396 | AF206495 | KR264332 | KR264366 | DQ360036 |
| *Meristogenys dyscritus* | AB360041 | AB526615 | --- | AB526670 | --- |
| *Meristogenys jerboa* | AB360044 | AB211493 | --- | AB360202 | --- |
| *Meristogenys orphnocnemis* | AB262545 | AB211494 | --- | EF088251 | DQ283847 |
| *Odorrana amamiensis* | AB200923 | AB200947 | --- | --- | --- |
| *Odorrana hosii* | AB200933 | AB200958 | KR264331 | EF088259 | KU840633 |
| *Odorrana livida* | DQ359969 | DQ650613 | --- | EF088260 | DQ360022 |
| *Odorrana schmackeri* | AB200935 | AB200959 | KX200497 | KX208784 | DQ360020 |
| *Odorrana supranarina* | AB200926 | AB200950 | --- | --- | --- |
| *Odorrana tormota* | EF453739 | EF453754 | --- | EU076750 | --- |
| *Odorrana utsunomiyaorum* | AB200928 | AB200952 | --- | --- | --- |
| *Pulchrana baramica* | AB719217 | DQ835353 | KR264314 | EF088265 | --- |
| *Pulchrana glandulosa* | AB719210 | EU604188 | --- | EF088270 | KU840647 |
| *Pulchrana laterimaculata* | AB719213 | EU604195 | --- | --- | --- |
| *Rana cascadae* | AY779197 | AY779197 | --- | KX269516 | --- |
| *Rana dalmatina* | AY043038 | AY147941 | --- | KX269563 | AY147992 |
| *Rana dybowskii* | DQ289100 | DQ289125 | --- | KX269553 | --- |
| *Rana forreri* | DQ283103 | DQ283103 | --- | KX269520 | DQ283818 |
| *Rana kukunoris* | NC_035804 | NC_035804 | --- | GQ285780 | GQ285798 |
| *Rana luteiventris* | AY779194 | AY779194 | --- | KX269578 | --- |
| *Rana okaloosae* | AY779203 | AY779203 | --- | --- | --- |
| *Rana palustris* | AY779228 | AY779228 | JN227143 | DQ347264 | DQ347383 |
| *Rana pyrenaica* | EU746401 | AY147950 | --- | KC798702 | AY148001 |
| *Rana septentrionalis* | AY779200 | AY779200 | --- | KX269529 | --- |
| *Rana sphenocephala* | DQ347040 | AY779252 | JN227096 | DQ347266 | DQ347385 |
| *Rana sylvatica* | DQ347052 | DQ283387 | --- | DQ019511 | DQ347397 |
| *Rana uenoi* | KX024861 | KX024887 | --- | --- | --- |
| *Rana vaillanti* | AY779214 | AY779214 | --- | KX269541 | --- |

**Rhacophoridae**

| **Species** | **12S** | **16S** | **CXCR4** | **RAG1** | **RHOD** |
| --- | --- | --- | --- | --- | --- |
| *Buergeria buergeri* | AB127977 | AB127977 | AB612035 | --- | --- |
