# Supplementary figures and images for: Noise constrains the evolution of call frequency contours in flowing water frogs: a comparative analysis in two clades
Source: Front Zool. 2021 Aug 4;18:37. doi: 10.1186/s12983-021-00423-y (PMC8336270; doi:10.1186/s12983-021-00423-y)

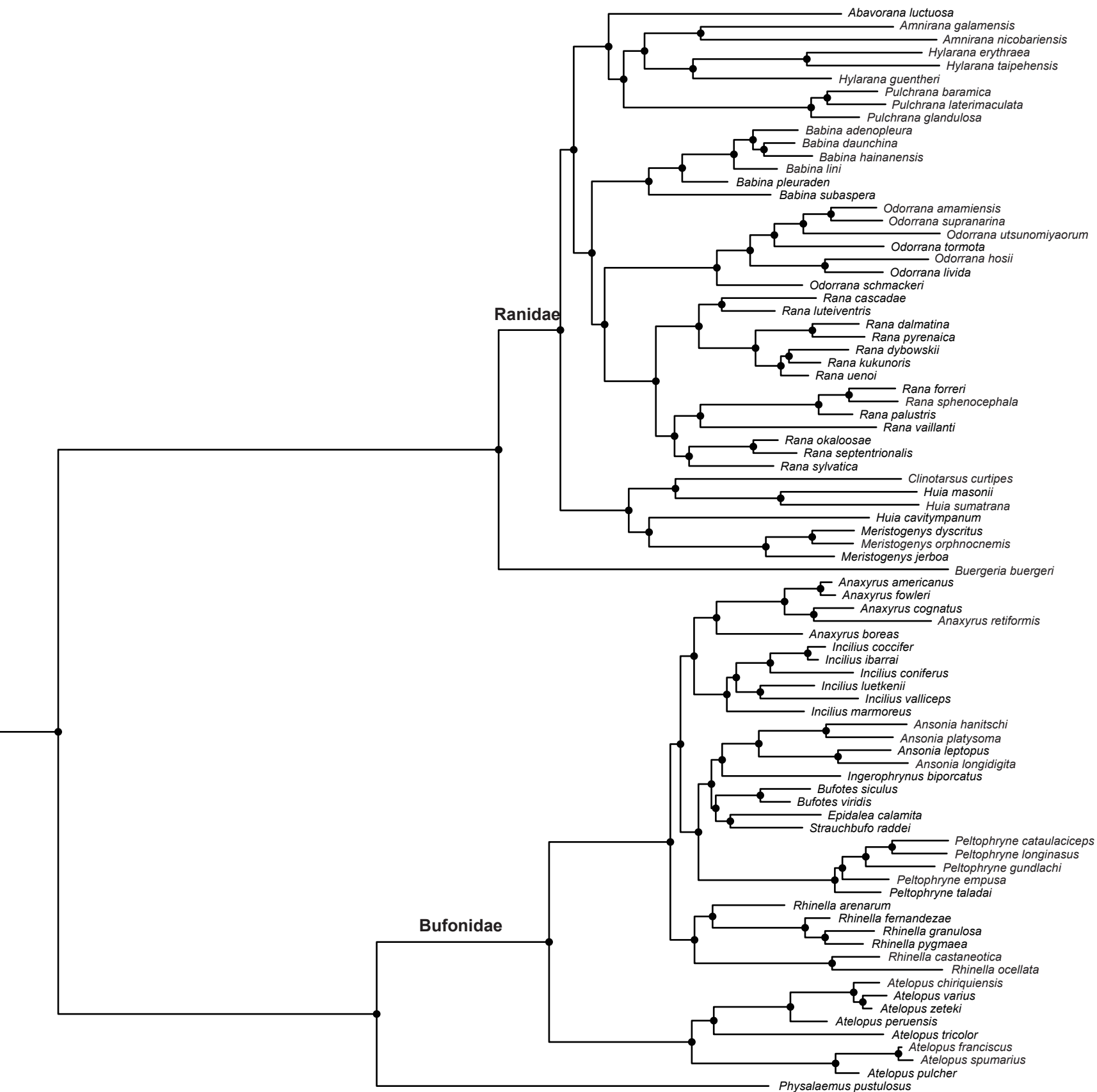

Supplement: Supplementary file 4 — Additional file 4: Figure S1. Phylogenetic relationships of all species based on two mitochondrial genes and three nuclear genes. [file 12983_2021_423_MOESM4_ESM.pdf]
